# Chaotic Mapping-Based Anti-Sorting Radio Frequency Stealth Signals and Compressed Sensing-Based Echo Signal Processing Technology

**DOI:** 10.3390/e24111559

**Published:** 2022-10-29

**Authors:** Jinwei Jia, Limin Liu, Yuying Liang, Zhuangzhi Han, Xuetian Wang

**Affiliations:** 1 Shijiazhuang Campus, Army Engineering University, Shijiazhuang 050003, China; 2 School of Aerospace Engineering, Nanchang Institute of Technology, Nanchang 330000, China; 3 School of Information and Electronics, Beijing Institute of Technology, Beijing 100081, China

**Keywords:** radio frequency stealth, anti-sorting, signal design, chaotic system, compressed sensing, signal processing

## Abstract

Radio frequency (RF) stealth anti-sorting technology can improve the battlefield survival rate of radar and is one of the research hotspots in the radar field. In this study, the signal design principle of anti-sequential difference histogram (SDIF) sorting was explored for the main sorting algorithm of the SDIF. Furthermore, we designed a piecewise linear chaotic system with interval number parameterization based on random disturbance and proposed a method to modulate the repetition period of widely spaced signal pulses using a chaotic system. Then, considering the difficulty of the traditional signal processing method to measure the velocity of the highly random anti-sorting signals designed in this paper, we used compressed sensing (CS) technology to process the echoes of the signals to solve the velocity and distance of the detection targets. Finally, simulation verification was performed from the correctness of the signal design principle, the performance of the chaotic system, the anti-sorting performance of the designed signals and the recovery and reconstruction performance of the signals by CS. The results show that: (a) the signal design principle presented in this paper can guide the signal design correctly; (b) the performance of the piecewise linear chaotic system with interval number parameterization is better than that of the classical one-dimensional chaotic system; (c) the anti-sorting signal modulated by the chaotic system can achieve anti-SDIF sorting, and the anti-sorting signals designed in this paper can be processed to obtain the velocity and distance of the targets.

## 1. Introduction

With the wide application of radar on the modern battlefield and the rapid development of signal processing technology, broadband real-time electronic reconnaissance equipment represented by radar warning receivers has rapidly improved the reconnaissance ability of radar; however, it greatly compresses the living space of radar on the battlefield and seriously affects the performance of radar [1,2,3,4]. Therefore, radio frequency (RF) stealth radar has become a research hotspot in the radar field. According to the three detection stages of signal interception, sorting and identification of broadband real-time electronic reconnaissance equipment such as radar warning receivers, the corresponding radar RF stealth mainly includes anti-interception [5,6,7], anti-sorting [8] and anti-identification. Anti-sorting, referring to preventing an electronic reconnaissance system from separating each radar pulse sequence from a random and interleaved pulse flow, is an important breakthrough point for anti-electronic reconnaissance systems [9].

Signal sorting is mainly divided into pre-sorting and main sorting. The pre-sorting means classifying pulses by their RFs, pulse widths (PWs) and directions of arrival (DOAs) to dilute a high-density pulse flow. The main sorting is the core of emitter signal sorting. It usually processes the time of arrival (TOA) of each pulse to obtain the pulse repetition interval (PRI) and the modulation mode of each emitter in the electromagnetic environment [10,11]. Therefore, to weaken the signal sorting of an electronic reconnaissance system, the first step is to combat the main sorting based on the TOA. Traditional RF stealth anti-sorting signal design techniques are mainly against PRI-based main sorting. There are three main methods to combat PRI-based main sorting. Firstly, pulse interference signals are added to radar signals to disturb the interception and identification of the PRI information of the pulse signal by an enemy intercept receiver [12,13,14,15], which is called jamming pulse anti-sorting. Secondly, a jitter is added to the PRI of each pulse signal to make the PRI different so that the enemy has difficulty intercepting the receiver to sort the radar signals [14,16,17], and we call this method jitter PRI anti-sorting. Thirdly, the signal PRI or the signal system is optimized with only the precise and quantitative design of the PRI, needing no additional interference pulse [18,19], which is called PRI optimization anti-sorting. Although the above methods have achieved certain results, they lack the comprehensive sorting failure principle as theoretical support. Therefore, the stability of the anti-sorting performance of the designed signals needs to be further verified. This paper shows that when signal PRI values change from being a fixed value to being in an interval distribution, and the length of the PRI interval distribution is greater than 20 times the minimum interval length between adjacent PRIs and the cumulant of the TOA difference histograms of the signal pulses at all levels is less than the threshold of the sorting algorithm. The sequential difference histogram (SDIF) algorithm fails to sort signals. In essence, it reduces the correlation between pulse trains and strengthens the randomness between them.

In order to ensure the high randomness of anti-sorting signal parameters, an improved chaotic algorithm is used to modulate the signal parameters. The chaotic system is the inherent randomness of a deterministic system and is a special motion in a nonlinear dynamic system [20]. Due to the typical characteristics, such as ergodicity, pseudo-randomness and initial sensitivity, chaos theory has important application value in chaotic image encryption and other fields [21,22,23,24]. With the development of chaos theory, its application scope has been gradually expanded from traditional cryptography, image encryption and other fields. Experts and scholars try to use chaotic systems to modulate the parameters of radar or communication signals. Yuxiao Yang [25] proposed a frequency-hopping system based on four-dimensional hyperchaos, in which the frequencies of communication signals were modulated by the chaotic system to achieve better RF stealth performance. Researchers realize that the complex dynamic behavior of chaos and hyperchaotic systems have a good application prospect in signal parameter modulation. Therefore, constructing a chaotic system that meets the real-time requirement of signal modulation and has high complexity is very important. Generally speaking, chaotic systems can be divided into one-dimensional chaotic systems and high-dimensional chaotic systems according to the number of variables in the systems. A one-dimensional chaotic system has only one variable, while a high-dimensional chaotic system has many variables [26,27,28]. Although a high-dimensional chaotic system has more unpredictability, complexity and sensitivity to initial values, it has some shortcomings, such as a large computation amount, complex structure and difficult realization. One-dimensional chaos is favored by researchers because of its simple chaotic structure and easy implementation. However, a simple chaotic structure inevitably leads to its limited chaotic interval, which is easy to be attacked by large-scale exhaustive algorithms. Therefore, the studies on chaotic systems have two main directions. One direction is that the randomness and complexity of chaotic systems can be enhanced by constructing high-dimensional chaotic systems. In 2014, 2015 and 2020, Peng Zaiping [29], Liu Yang [30] and Yang Yuxiao [25] respectively proposed four-dimensional hyperchaotic systems. Three kinds of hyperchaotic systems were constructed by adding a new state feedback controller based on a three-dimensional chaotic system. During the construction of each four-dimensional hyperchaotic system, the newly added controller must be fed back to the original controller, and the original controller must be fed back to the new controller. These two operations can make the four controllers of the system interact with each other, complicating the relationship. However, whether in the hardware implementation or simulation stage, the four-dimensional chaotic system requires a high computing platform, and the operation time is long. In signal modulation and other fields, it is desirable to use the strong randomness of high-dimensional chaotic systems and the high time requirement. Therefore, Jia Jinwei [31] designed a two-dimensional interleaving feedback chaotic system based on cosine exponents. The designed chaotic system was used to modulate multiple signal parameters, and the composite modulation of signal parameters was realized. The anti-sorting ability of the signals was improved. The other direction is that it is an improvement of the classical one-dimensional chaotic system, making the improved one-dimensional chaotic system more complex and stochastic. Dao Xinyu [32] and Jia Jinwei [33] designed improved one-dimensional chaotic systems based on cosine and exponential functions. Compared with the classical one-dimensional chaotic system, the improved one-dimensional chaotic systems have been significantly improved in terms of randomness, complexity and the balance of the generated chaotic sequences, and can better meet the needs of the application scenarios using chaotic systems to modulate radar signal parameters.

A chaotic signal has strong randomness, which can meet the requirements of the anti-sorting signal. However, it brings great difficulties to echo signal processing. For the processing of highly random signals, the correlation method [34], spectrum method [35], inverse correlation method [35] and compressed sensing (CS) [36,37] are the main methods at present. Among them, CS has the characteristics of non-uniform sampling, a small data amount, fast processing speed, high accuracy of signal reconstruction and recovery, and great advantages in random signal processing. Therefore, CS technology is used to process anti-sorting signals in this paper.

In 2006, David Donoho, Emmanuel Candes, and Terence Tao proposed CS, in which a set of random projections are used to sense the original signal at much lower than the Nyquist sampling rate [38]. The dimension of the observed data is greatly reduced, and then the original signal is reconstructed using the sparsity characteristic of the signal. Using CS theory, the amount of sampling data is greatly reduced to realize broadband signal processing. In recent years, CS theory has been fully applied in signal processing. Zhang Qinghe [39] used CS technology to conduct microwave imaging research on different scatterers, which improved the imaging accuracy and reduced the imaging error. Li Jiaqiang [40] combined CS with radar imaging, constructed a two-dimensional sparse representation model of the range-frequency domain and azimuth Doppler domain through radar echoes, and transformed the imaging problem into a sparse reconstruction model of CS in the range direction and azimuth direction. Du Siyu [41] and Sui Jinping [42] applied CS theory to process target echo signals, extracted the distance and velocity information of the real targets in the signals, and achieved radar anti-jamming. In summary, CS theory is especially suitable for solving the sampling problem and storage pressure of large-bandwidth signals and large-capacity data due to its compressed observation characteristics, and it is widely used in radar signal processing, image processing, electronic countermeasures and other fields.

In this study, the workflow of the main sorting algorithm based on the SDIF algorithm was analyzed, the failure principle of the SDIF algorithm was studied, and the failure principle was selected as the signal design principle for anti-SDIF sorting. Using the chaotic system with interval number parameterization designed in this paper, the RF stealth anti-sorting signals were designed, and the anti-sorting performance of the signals was verified by simulation. The structure of the whole paper is as follows. In Section 2, the failure principle of SDIF sorting is analyzed in detail. Section 3 shows a segment number parameter-piecewise linear chaotic map (SNP-PLCM) based on random disturbance and widely spaced PRI pulse signals designed based on the SNP-PLCM. In Section 4, according to the characteristics of the anti-sorting signals, a CS dictionary based on chaos theory is proposed to reconstruct the anti-sorting signals designed in Section 3 by velocity-distance two-dimensional restoration and reconstruction and obtain the velocity and distance parameter information of the targets. Section 5 involves the simulation and verification of three or four parts of SNP-PLCM performance, the anti-sorting performance of the designed signals, detection performance and the CS for echo signal processing. Section 6 is a summary of the full text.

## 2. Principle of Anti-Sorting Signal Design

The research on signal sorting algorithms indicates that the principle of anti-sorting signal design is the failure mechanism of signal sorting. Therefore, the signal sorting failure mechanism provides theoretical support for designing anti-sorting signals, thus improving the efficiency and success rate of anti-sorting signal design.

### 2.1. Sorting Algorithm Based on the SDIF

Radar signal source sorting is also known as the deinterleaving of radar radiation source signals. It refers to the process of separating radar pulse trains from a random and interleaved pulse flow. In essence, it is the parameter matching of each signal pulse. In engineering, histogram sorting is the most commonly used method to estimate the PRI value of radiation source signals based on the statistical principle. After the differences in TOA are counted, the histogram of the difference is formed. Then, the appropriate sorting threshold and sorting strategy are set. The cumulative difference histogram (CDIF) and SDIF are two improved algorithms commonly used in engineering.

Both the SDIF and CDIF are TOA difference histogram sorting methods. The TOA differences of pulses are counted by the two sorting algorithms according to certain rules, and PRI estimation is analyzed. Then, the impulse sequence search is performed based on the PRI estimation. Lastly, the radiation source pulse trains are extracted. Compared with the traditional histogram sorting algorithm, the SDIF and CDIF algorithms greatly reduce the computation amounts and have real-time performance. The SDIF and CDIF algorithms widely used in engineering can be used to sort fixed PRI, staggered PRI and jitter radiation source signals.

Compared with the CDIF algorithm, the SDIF algorithm has the following advantages. In the SDIF, only the histograms of the current level are analyzed, and the histogram statistics of different levels and the two PRI tests are not performed. It requires a lesser computation amount and has a higher processing speed. In addition, the SDIF algorithm has an optimized threshold function. By combining the SDIF algorithm with subharmonic detection, false detection can be avoided. Therefore, the SDIF is more widely used than the CDIF. The flows of the SDIF algorithm are as follows.

According to Algorithm 1, the SDIF mainly includes TOA difference histogram analysis and impulse sequence search. The TOA difference histogram analysis is used to estimate PRI values. The histogram statistics method is used to calculate the number of TOA differences from one level to a higher level. If the number of TOA differences exceeds the detection threshold, the TOA difference corresponding to the peak divided by the statistical series of the TOA difference is the possible PRI value. The threshold function of the SDIF can be expressed as:

(1) $$T_{thre}\left(\tau \right)=a\left(E-C\right)e^{-\tau /kN}$$

where *E* is the total number of pulses, *C* is the order of the histogram, k is a positive constant less than 1, *N* is the total number of bins in the histogram, *a* is an adjustable constant and τ is the PRI value. The optimum values of the constants *a* and *k* are experimentally determined.


**Algorithm 1: Deinterleaving signals via the SDIF**
Input: TOAInitialization: The order of the histogram *C*1: Judge the TOA number of level *C*2: Calculate the TOA difference of level *C*3: Count the TOA difference of level *C* and form a histogram4: Determine the detection threshold and obtain the PRI estimation5: Check subharmonics6: Determine the unique PRI estimation7: Perform sequence search8: Remove the searched pulse train from the pulse flow9: Sort the remaining pulses until the number of pulses is less than five or the number of PRIs in the first-level histogram exceeding the threshold is not unique10: Increase difference level *C* and repeat the above steps until the sorting is over

In the actual signal sorting process by the SDIF sorting algorithm, the influence of the intercept receiver on pulse TOA measurement should be avoided, and the sorting performance of the algorithm for jitter signals should be enhanced. Therefore, the tolerance ε of PRI values τ is set in the SDIF and its improved sorting algorithm. The interval of the signal PRI value τ, i.e., the PRI interval or a small box of PRI, is determined by the tolerance. The upper and lower limits of the small box can be expressed as:

(2) $$\tau_{\max}=\tau +\varepsilon$$

(3) $$\tau_{\min}=\tau -\varepsilon$$

Then, the box range of the PRI value is $\tau_{\min}\leq \tau \leq \tau_{\max}$. The PRI value of the histogram is the weighted average of the values that fall into the PRI box. Hence, the weighted average function should be:

(4) $$\bar{\tau }=\sum_{i=1}^{n}\left(x_{i}/S\right)\cdot \tau_{i}$$

where S is the sum of the pulse numbers corresponding to adjacent PRI values $\tau_{1},\tau_{2},\cdot \cdot \cdot ,\tau_{i}$ within the tolerance, and $x_{i}$ is the number of PRI values $\tau_{i}$ corresponding to pulses within the tolerance.

### 2.2. Sorting Failure Principle

#### 2.2.1. Analysis of the Sorting Failure Principle of the First-Order Histogram

When the signals are fixed-period signals, i.e., $\tau =\tau_{0}$, the number of pulses *E* is assumed to be $E_{0}$; $e^{-\frac{\tau }{kN}}$ and *a* are positive constants less than 1 when τ>0 in the threshold Equation (1). The number of pulses is $E_{0}\gg 1$ in the actual situation. Therefore, the right part of the threshold equation is $a\left(E_{0}-C\right)e^{-\frac{\tau }{kN}}<E_{0}$, and the threshold equation is $T_{thre}\left(\tau_{0}\right)<E_{0}$ when $\tau =\tau_{0}$. The signal PRI value $\tau =\tau_{0}$ can be sorted out.

① The number of PRI values increases from one to a finite number.

Assuming the total number of pulses $E=E_{0}$ is constant, the signals are two staggered signals when the number of PRI values increases from one to two. In addition, the PRI value is $\tau_{0}$ or $\tau_{1}$ ($\tau_{0}<\tau_{1}$). There are $\frac{E_{0}}{2}$ pulses in each PRI value. The relationship between the number of corresponding pulses and the threshold $T_{thre}\left(\tau_{0}\right)$ is discussed when the PRI value and pulse number change.

According to Equation (1), when the PRI value is $\tau =\tau_{0}$, the threshold of the algorithm should be:

(5) $$T_{thre}\left(\tau_{0}\right)=a\left(E_{0}-1\right)e^{-\tau_{0}/kN}$$

When the PRI value is $\tau =\tau_{1}$, the threshold of the algorithm can be expressed as

(6) $$T_{thre}\left(\tau_{1}\right)=a\left(E_{0}-1\right)e^{-\tau_{1}/kN}$$

When the threshold of the algorithm is $\frac{E_{0}}{2}$, i.e., $T_{thre}\left(\tau^{\prime }\right)=\frac{E_{0}}{2}$, the PRI critical value of sorting failure can be calculated by Equation (7).

(7) $$a\left(E_{0}-1\right)e^{-\tau^{\prime }/kN}=\frac{E_{0}}{2}$$

where $E_{0}$ represents the total number of pulses (usually in the tens of thousands), i.e., $E_{0}\gg 1$. Therefore, Equation (7) can be simplified as:

(8) $$aE_{0}e^{-\tau^{\prime }/kN}=\frac{E_{0}}{2}$$

After taking the natural logarithm of the above equation, $\tau^{\prime }$ can be obtained:

(9) $$\tau^{\prime }=kN\ln\left(2a\right)$$

According to the monotonically decreasing property of the threshold function, it can be known that the SDIF algorithm can sort the pulses of PRI $\tau_{0}$ when $\tau_{0}\geq kN\ln\left(2a\right)$, i.e., $\frac{E_{0}}{2}\geq T_{thre}\left(\tau_{0}\right)$. The SDIF algorithm cannot sort the pulses of PRI $\tau_{0}$ when $\frac{E_{0}}{2}<T_{thre}\left(\tau_{0}\right)$. So, if $\tau_{1}\geq kN\ln\left(2a\right)$, the SDIF algorithm can sort the pulses of PRI $\tau_{1}$, and if $\tau_{1}<kN\ln\left(2a\right)$, the SDIF algorithm cannot sort them. Consequently, the SDIF algorithm cannot sort the signal pulses of which the PRI is less than the critical value kNln(2a) when the number of PRI values increases from one to two.

According to the above analysis, the actual signal sorting situation is extended, and the derivation condition is assumed as follows:

(1) The total number of pulses is still $E_{0}$
(2) The number of PRI values increases to a finite number, meaning that the signals are multiple staggered signals.
(3) The PRI values are $\tau_{1}$, $\cdot \cdot \cdot \tau_{n}$($\tau_{1}<\cdot \cdot \cdot <\tau_{n}$).
(4) The number of pulses corresponding to each PRI value is $\frac{E_{0}}{n}$.

The derivation process is the same as that of Equations (5)–(9). Hence, the derivation will not be repeated here. The PRI critical value of sorting failure is solved as:

(10) $$\tau^{\prime }=kN\ln\left(na\right)$$

Finally, the SDIF algorithm cannot select the signal pulses of which the PRI is less than the critical value kNln(na) when the number of PRI signals increases to a finite number.

② The signal PRI values follow an interval distribution.

It is assumed that the number of pulses *E* is $E_{0}$. The signal PRI values follow an interval distribution $\tau \in \left[\tau_{0},\tau_{1}\right]$. The design of transmitting signals and the processing of echo signals need to be considered in the radar field. The PRI values of radar signals do not follow a completely random disordered distribution. The PRI values of signals discussed in this section follow a uniform distribution. For any given interval, the number of pulses $E^{\prime }$ is:

(11) $$E^{\prime }=\frac{E_{0}}{\left(\tau_{1}-\tau_{0}\right)/z}$$

where $E_{0}$ is the total number of pulses, $\tau_{1}$ and $\tau_{0}$ are the maximum and minimum values of the distribution intervals, and *z* is the minimum interval of PRI values within the interval distribution. When the PRI is $\tau^{\prime }$, the threshold calculation equation of the algorithm is shown in Equation (1).

When $T_{thre}\left(\tau^{\prime }\right)=E^{\prime }$,

(12) $$a\left(E_{0}-1\right)e^{-\tau^{\prime }/kN}=\frac{E_{0}}{\left(\tau_{1}-\tau_{0}\right)/z}$$

where $E_{0}$ is the total number of pulses, and $E_{0}\gg 1$. Therefore, the above equation can be simplified as:

(13) $$aE_{0}e^{-\tau^{\prime }/kN}=\frac{E_{0}}{\left(\tau_{1}-\tau_{0}\right)/z}$$

Then, the PRI critical value of sorting failure is solved as:

(14) $$\tau^{\prime }=kN\ln\left(\frac{a\cdot \left(\tau_{1}-\tau_{0}\right)}{z}\right)$$

When $\tau_{1}<\tau^{\prime }$, the number of pulses corresponding to PRI values in the interval $\left[\tau_{0},\tau_{1}\right]$ is less than the threshold. Hence, the SDIF algorithm sorting fails. When $\tau_{0}<\tau^{\prime }<\tau_{1}$, the number of pulses corresponding to the PRI values in the interval $\left[\tau_{0},\tau^{\prime }\right)$ is less than the threshold. Therefore, the SDIF algorithm sorting also fails in the interval $\left[\tau_{0},\tau^{\prime }\right)$. When $\tau_{0}<\tau^{\prime }<\tau_{1}$, the number of pulses corresponding to the PRI values in the interval $\left[\tau_{0},\tau^{\prime }\right)$ is greater than the threshold. The signals can be sorted successfully. When the PRI values of the signals follow the interval distribution, the SDIF algorithm cannot select the signal pulses of which the PRI is less than the critical value $kN\ln\left(\frac{a\left(\tau_{1}-\tau_{0}\right)}{z}\right)$.

The conditions in the actual signal sorting process are described as follows:

(1) *N* is the number of cells counted in the histogram, which is usually above 1000.
(2) *z* is the minimum interval of PRI values. In general, z, $\tau_{1}$, and $\tau_{0}$ have the same size scale and all are at the microsecond level.
(3) a and k ∈(0,1) in Equation (1).

Therefore, when the interval length is 20 times larger than *z*, we can know $\ln\left(\frac{a\cdot \left(\tau_{1}-\tau_{0}\right)}{w}\right)>1$. The threshold is much larger than any value in the interval $\left[\tau_{0},\tau_{1}\right]$, i.e., $\tau^{\prime }\gg \tau_{1}$.

In conclusion, when signal PRI values follow the interval distribution, and the interval length is 20 times larger than *z*, the number of pulses corresponding to any PRI value in the interval $\left[\tau_{0},\tau_{1}\right]$ is less than the threshold of the sorting algorithm in the analysis of the sorting failure principle of the first-order histogram. Hence, the SDIF algorithm fails in signal sorting.

#### 2.2.2. Analysis of the Sorting Failure Principle of the Multi-Order Histogram

Sometimes, the SDIF sorting algorithm has multiple PRI values exceeding the threshold in the first-order histogram. It is necessary to count the second-order, third-order, and higher-order histograms to produce PRI estimation. Furthermore, the sorting failure principle of the second-order, third-order, and higher-order histograms also needs to be analyzed.

The threshold function is shown in Equation (1). In the second-order to multi-order histograms, only the histogram order *C* changes. The total number of pulses *E* is much larger than C, i.e., E≫C.

Thus, the threshold function is approximately unchanged. When the signal PRI values follow the interval distribution, the first-order histogram in Section 2.2.1 is still applicable to the analysis of the sorting failure principle of the SDIF algorithm.

In summary, when the PRI values of the radar signals follow the interval distribution of which the length is more than 20 times the minimum interval of PRI values, the accumulation of signal pulse difference histograms at all levels is less than the threshold of the sorting algorithm, leading to its failure.

### 2.3. Principles of Signal Design

The essence of signal sorting is to use the correlation between pulses in the pulse flow to “match” the pulse sequences belonging to the same radiation source with each other to realize signal sorting.

According to the sorting failure principle in Section 2.2, when the PRI values of the radar signals follow the interval distribution of which the length is more than 20 times the minimum interval of the PRI values, the cumulative amount of the signal pulses in the TOA difference histogram at all levels is smaller than the threshold of the sorting algorithm. Therefore, the SDIF sorting algorithm fails. The principle of sorting failure displays the essence of RF stealth signal design, which is to reduce the correlation between pulse sequences and strengthen the randomness between pulses. The signal sorting algorithm is designed for thousands of approximately disordered random pulse flows. Therefore, not any random pulse sequence with low correlation has resistance to sorting, which requires targeted design based on the failure principle of SDIF sorting. Chaotic sequences have the characteristics of strong randomness. Therefore, according to the failure principle of SDIF sorting, we proposed a method for designing anti-sorting signals based on the SNP-PLCM chaotic system using the chaotic sequence.

## 3. Anti-Sorting Signal Design of the SNP-PLCM Chaotic System Based on Random Disturbance

### 3.1. Construction of the SNP-PLCM Chaotic System Based on Random Disturbance

Equation (15) is the classical Tent map.

(15) $$x_{n+1}=\{\begin{array}{ll} \mu x_{n}, & 0\leq x_{n}<0.5\\ \mu \left(1-x_{n}\right), & 0.5\leq x_{n}<1 \end{array}$$

In Equation (15), μ is the fractal coefficient of the Tent mapping. $x_{n}$ is the value of the chaotic mapping at this moment, and $x_{n+1}$ is the iteration value of the chaotic mapping at the next moment. By introducing the interval number selection parameter and interval control coefficient and using mathematical methods, the interval number of the chaotic map is parameterized to generate a multi-segment PLCM. The chaotic map with different interval numbers is selected according to different application scenarios. The expression of the chaotic map is shown in Equation (16).

(16) $$x_{n+1}=f\left(x_{n},\mu \right)=\left\{ \begin{array}{l} \begin{array}{cc} \frac{x_{n}-i\mu }{\frac{\mu }{l}}, & \frac{i\mu }{l}\leq x_{n}\leq \frac{\left(i+1\right)\mu }{l}\\ \frac{x_{n}-\left(\mu +\frac{i\left(0.5-\mu \right)}{l}\right)}{\frac{0.5-\mu }{l}}, & \mu +\frac{i\left(0.5-\mu \right)}{l}\leq x_{n}\leq \mu +\frac{\left(i+1\right)\left(0.5-\mu \right)}{l} \end{array}\\ \begin{array}{cc} x_{n}+r_{s}, & x_{n}=0.5\\ \begin{array}{c} f\left(1-x_{n},\mu \right),\\ \left|x_{n}\right|+r_{s}, \end{array} & \begin{array}{c} 0.5<x_{n}<1\\ x_{n}<0 \end{array} \end{array} \end{array}\right.$$

**Figure 1:**
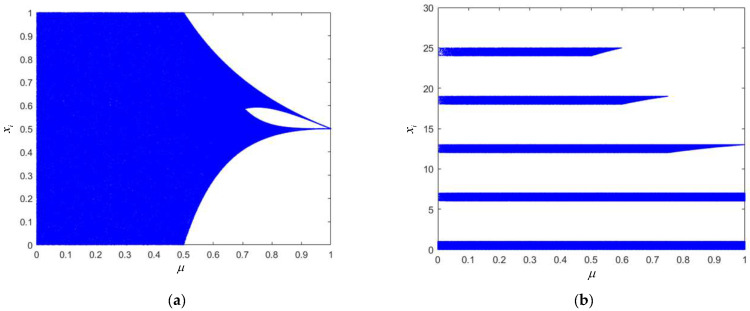
Bifurcation diagrams of the chaotic mappings designed in this paper. (**a**) Bifurcation diagram of the SNP-PLCM mapping with 4 intervals (interval number selection parameter *l* = 1). (**b**) Bifurcation diagram of the SNP-PLCM mapping with 24 intervals (interval number selection parameter *l* = 6).

**Figure 2:**
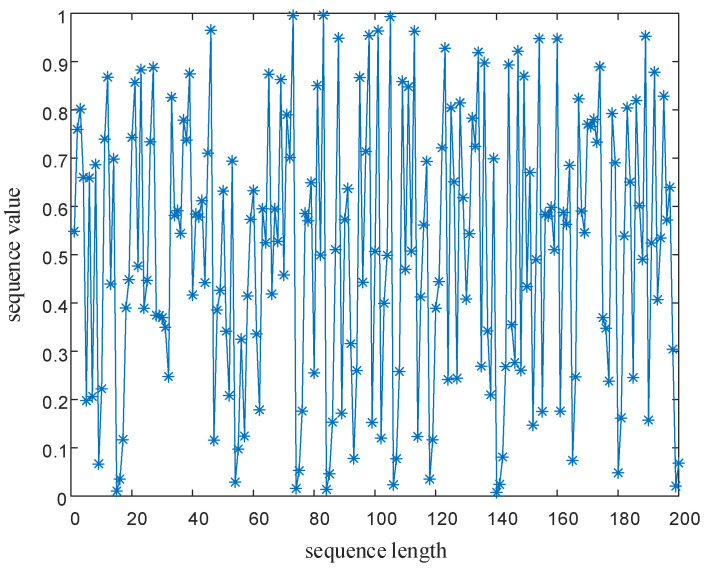
Point diagram of the chaotic mapping sequence designed in this paper.

In Equation (16), μ is the fractal coefficient, i is the interval control coefficient, and l is the interval number selection parameter. The number of segment intervals of the mapping is 4⋅l, and $r_{s}$ is the random disturbance of the chaotic mapping. Based on the classic Tent mapping, the interval number parameter is introduced to parameterize the interval number. The interval number is no longer a fixed two or four and is beneficial to the combination of the chaotic mapping and different application cases. Compared with other chaotic maps, the map designed in this paper has few control parameters, which is convenient for the realization of software and hardware. If the control parameters of the chaotic map are used as the key, the key can be managed conveniently. Thus, the speed problem of encryption or signal modulation can be solved to a large extent. Moreover, the key can even be applied to real-time modulation or real-time encryption systems. Additionally, a random disturbance signal is coupled into the chaotic map at $x_{n}=0.5$ and $x_{n}<0$, so that the chaotic map can overcome the short-period effect caused by limited calculation accuracy. Certainly, interval parameterization can also be used to overcome short-period effects. The bifurcation diagrams of the chaotic mappings and the point diagram of the chaotic mapping sequence designed in this paper are shown in Figure 1 and Figure 2.

**Figure 3:**
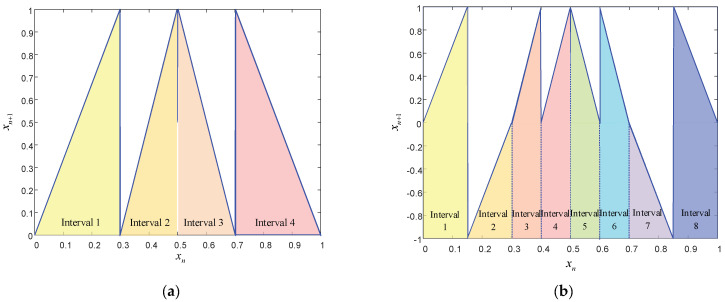
The interval segmentation diagrams of the chaotic mappings designed in this paper. (**a**) Chaotic mapping with 4 intervals (interval number selection parameter *l* = 1). (**b**) Chaotic mapping with 8 intervals (interval number selection parameter *l* = 2).

The bifurcation diagram in Figure 1a shows that when *l* = 1, the system is in a chaotic state when the fractal coefficient μ∈(0,0.5], and the system is in a periodic state when the fractal coefficient μ∈(0.5,1). As shown in Figure 1b, when *l* = 6, the chaotic map proposed in this paper is not affected by the fractal coefficient, and the points are relatively uniformly distributed in the bifurcation diagram. There are five distribution sets of chaotic sequence values, including $x_{n}\in \left(0,1\right)$, $x_{n}\in \left(6,7\right)$, $x_{n}\in \left(12,13\right)$, $x_{n}\in \left(18,19\right)$ and $x_{n}\in \left(24,25\right)$ in the bifurcation diagram, expanding the hopping range of chaotic sequence points and enhancing the encryption or modulation performance of the chaotic mapping. During actual encryption or modulation, different ranges of chaotic sequence point values can be used to encrypt pixels at different positions of the image or modulate different signal parameters, and one-dimensional chaotic mapping can be used to achieve multi-dimensional modulation. As shown in the interval segmentation diagrams in Figure 3, the interval numbers of the chaotic map designed in this paper increase by 4·l with the increase in the interval number selection parameter *l*.

### 3.2. Method for Designing Widely Spaced Signals

The signal parameters are modulated according to the sequence generated by the SNP-PLCM. According to Section 2, the SDIF algorithm is to sort the PRI values of radar signals, so the signal PRI values T are modulated by the chaotic sequence. In order to further enhance the anti-sorting performance of the signals to enable them to resist clustering and sorting, different sequence value sets of the chaotic map are used to modulate the carrier frequency value F of the signals. The modulation function is shown as Equations (17) and (18).

(17) $$T_{n}=T_{0}+\left(x_{n1}-0.5\right)\cdot \Delta T\;x_{n1}\in \left(0,1\right)$$

(18) $$F_{n}=F_{0}+\left(x_{n2}-6.5\right)\cdot \Delta F\;x_{n2}\in \left(6,7\right)$$

**Figure 4:**
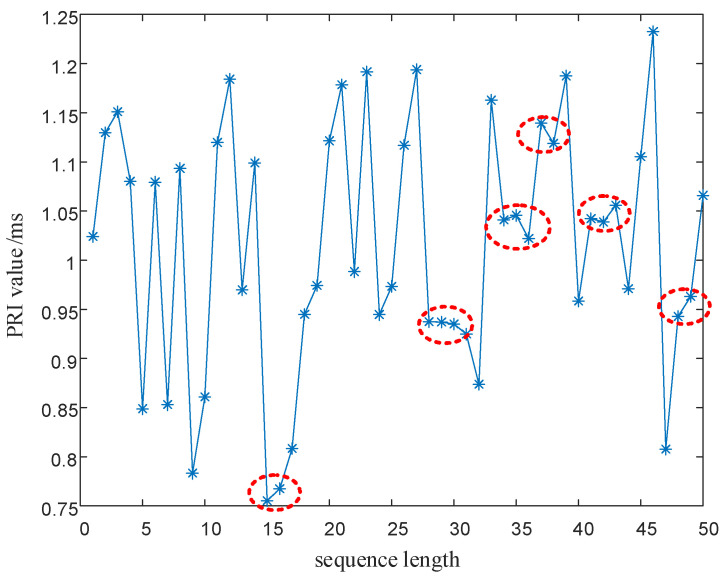
Plot of the multi-value narrow interval effect after PRI modulation.

**Figure 5:**
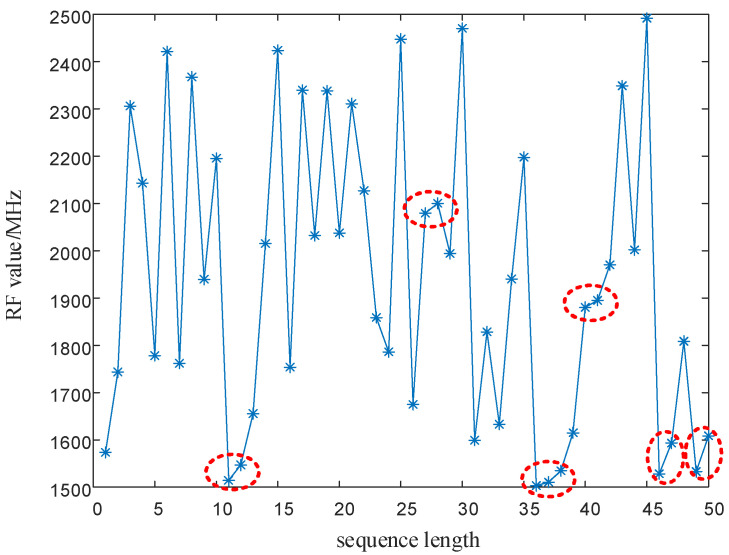
Plot of the multi-value narrow interval effect after RF modulation.

In Equation (17), $T_{0}$ is the PRI center value of the signal, ΔT is the maximum variation of the signal PRI values, $F_{0}$ is the RF center value of the signal, and ΔF is the maximum variation of the signal RF values. $x_{n1}$ is generated by the SNP-PLCM of Equation (16), and the PRI value T is modulated by the point set of the (0,1) sequence. $x_{n2}$ is generated by the SNP-PLCM of Equation (16), and the carrier frequency value F is modulated by the point set of the (6,7) sequence. The modulated PRI value and RF value sequences still have the randomness of the chaotic sequence. However, as shown by the red circles in Figure 4 and Figure 5, the PRI value and RF value sequences modulated by the chaotic map have multi-value narrow interval effects. That is, there are multiple adjacent pulses in the sequence with small intervals between PRI values, causing adjacent pulses to fall within the same pulse sorting tolerance. Likewise, the multi-value narrow interval effects of the PRI value and RF value sequences can also reduce the anti-cluster sorting performance of the signals.

Therefore, when using the chaotic mapping for modulation, it is necessary to improve the generation method of chaotic sequences to eliminate the multi-value narrow interval effects due to the direct modulation of the chaotic sequence.

**Figure 6:**
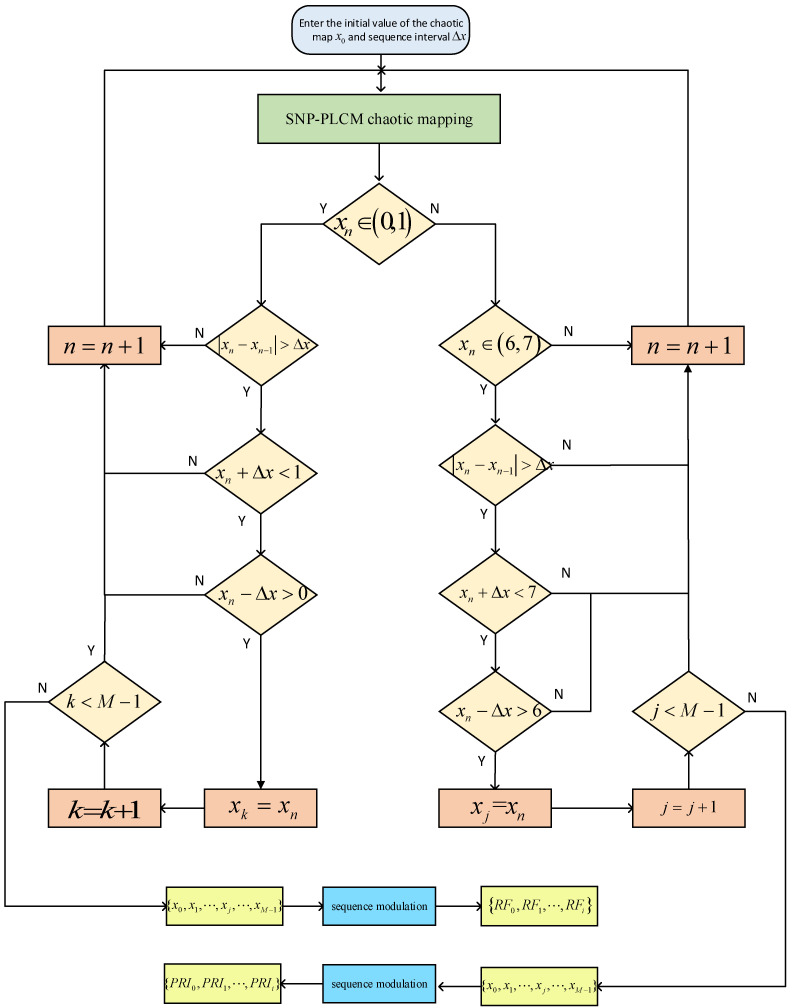
Flowchart of the modulation of signal PRI and RF sequences.

Each time a sequence value $x_{n}$ is generated by the chaotic map, it is firstly classified into the corresponding (0,1), (6,7) or other sequence point sets. Then, whether the interval between the two sequence values of $x_{n}$ and the previous sequence value $x_{n-1}$ in each sequence point set meets the interval requirement is judged. If the interval requirement is met, the value $x_{n}$ is kept and substituted into the chaotic map expression, the next iteration is performed to generate $x_{n+1}$, and then the interval between $x_{n+1}$ and $x_{n}$ is judged. If the interval does not meet the requirement, $x_{n}$ is discarded, and whether the interval between $x_{n-1}$ and the newly generated $x_{n+x}$ meets the requirement is judged. If the interval requirement is met, $x_{n+x}$ is kept. If the interval requirement is not met, $x_{n+x}$ is discarded, and the interval between $x_{n-1}$ and the sequence value assigned to the corresponding point set is compared until the chaotic sequence value that meets the requirement is obtained. Finally, the signal PRI values are modulated by the chaotic sequence values meeting the conditions according to modulation Equations (17) and (18). Figure 6 intuitively displays the whole process of modulation and chaotic sequence value screening.

## 4. Anti-Sorting Signal Processing Based on CS

According to the analyses in Section 2 and Section 3, the PRI and RF values of the anti-sorting signals designed in this paper are in an irregular jump state. Traditional signal processing technology cannot process PRI and RF multi-parameter composite modulation signals, so CS technology is needed. In this section, a multi-parameter composite modulated signal model is firstly established, and then a CS dictionary matrix based on chaotic sequences is constructed according to the signal model. Finally, CS technology is used to recover and reconstruct the echo signals and calculate the distance and velocity of the targets.

### 4.1. Multi-Parameter Composite Modulated Signal Model

**Figure 7:**
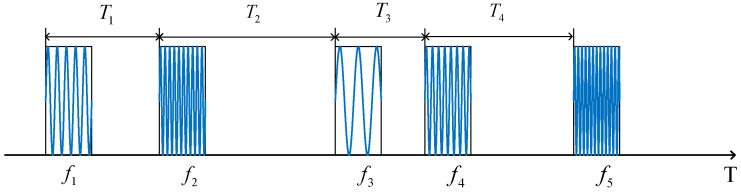
Multi-parameter composite modulated signal model with 5 pulses in a coherent processing interval (CPI) taken as an example.

The multi-parameter composite modulated signal model is shown in Figure 7.

It is assumed that the radar transmits *W* pulses in a CPI, and the carrier frequencies of any two pulses are modulated by the chaotic mapping within the frequency variation range $\left(f_{1},f_{2}\right)$. Assuming that the frequency of the a-th transmitted pulse is $f_{a}$, according to Equation (19),

(19) $$f_{a}=f_{0}+\left(x_{n2}-6.5\right)\cdot \Delta f\;x_{n2}\in \left(6,7\right)\;n2\in \text{\{}1,2,\cdot \cdot \cdot ,W\text{\}}$$

$f_{0}$ is the RF center value of the signal, and Δf is the maximum variation of the signal RF values. $x_{n2}$ is generated by the SNP-PLCM in Equation (16), and the carrier frequency value F is modulated by the point set of the (6,7) sequence, and n2 is the total number of chaotic sequences. Therefore, the a-th transmitted signal model can be expressed as:

(20) $$S_{a}\left({\hat{t}}_{a}\right)=rect\left(\frac{{\hat{t}}_{a}}{T_{p}}\right)\cdot \exp\left(j2\pi f_{a}{\hat{t}}_{a}\right)\cdot \exp\left(\phi_{a}\left({\hat{t}}_{a}\right)\right)$$

In Equation (20), *a* is the serial number of the transmitted pulse in a CPI, where a∈[1,2,⋅⋅⋅,W]. $T_{p}$ is the PW of the transmitted signal. ${\hat{t}}_{a}$ is the fast time of a signal pulse, and $0<{\hat{t}}_{a}<T_{p}$. $\phi_{a}\left({\hat{t}}_{a}\right)$ is the phase of the transmitted signal.

In the practice field of radar engineering, to obtain high-range resolution, linear frequency modulation (LFM) signals are usually designed, among which the LFM signal is the most widely used. In combination with the specific situation of signal design in Section 3, the LFM signal is studied in this section. The transmitted signal model of radar is:

(21) $$S_{a}\left({\hat{t}}_{a}\right)=rect\left(\frac{{\hat{t}}_{a}}{T_{p}}\right)\cdot \exp\left(j2\pi f_{a}{\hat{t}}_{a}\right)\cdot \exp\left(j\pi K{\hat{t}}_{a}^{2}\right)$$

In Equation (21), *K* is the modulation frequency of the LFM signal, $K=\frac{B}{T_{p}}$, and *B* is the bandwidth of the LFM signal. M targets are assumed within the detection range of the radar, the distance between each target and the radar is $r_{m}$, and the radial velocity of each target relative to the radar is $v_{m}$, where *m* is the target serial number, m∈[1,M]. Therefore, in a CPI, the echo of the a-th signal pulse is:

(22) $$S_{a}\left({\hat{t}}_{a}\right)=\sum_{m=1}^{M}rect\left(\frac{{\hat{t}}_{a}-\tau_{m}}{T_{p}}\right)\cdot \exp\left(j2\pi f_{a}\left({\hat{t}}_{a}-\tau_{m}\right)\right)\cdot \exp\left(j\pi K{\left({\hat{t}}_{a}-\tau_{m}\right)}^{2}\right)$$

In Equation (22), the time delay of the m-th target is:

(23) $$\tau_{m}=2\frac{r_{m}+v_{m}T_{a}}{c}$$

In Equation (23), $T_{a}$ is the slow time series. According to Equation (17), it can be known that:

(24) $$T_{a}=T_{0}+\left(x_{n1}-0.5\right)\cdot \Delta T\;x_{n1}\in \left(0,1\right)\;n1\in \{1,2,\cdot \cdot \cdot ,W\}$$

$T_{0}$ is the PRI center value of the signal, and ΔT is the maximum variation of the signal PRI values. $x_{n1}$ is generated by the SNP-PLCM in Equation (16), and the PRI value T is modulated by the point set of the (0,1) sequence, and n1 is the total number of chaotic sequences. To ensure that the frequencies of any two pulses among *W* pulses in a CPI are different, the total number of chaotic sequences is at least greater than *W*.

After receiving the echo signal, the radar receiver mixes the echo at the corresponding transmitting frequency point, and the radar echo signal after mixing the frequency is expressed as:

(25) $$S_{a}\left({\hat{t}}_{a}\right)=\sum_{m=1}^{M}rect\left(\frac{{\hat{t}}_{a}-\tau_{m}}{T_{p}}\right)\cdot \exp\left(-j2\pi f_{a}\tau_{m}\right)\cdot \exp\left(j\pi K{\left({\hat{t}}_{a}-\tau_{m}\right)}^{2}\right)$$

Pulse compression is performed on the echo signal after down-conversion, and the echo signal after pulse compression is expressed as:

(26) $$S_{a}\left({\hat{t}}_{a}\right)=\sum_{m=1}^{M}A_{m}\cdot \exp\left(-j2\pi f_{a}\tau_{m}\right)\cdot \sin c\left(\pi B\left({\hat{t}}_{a}-\tau_{m}\right)\right)$$

In Equation (26), $A_{m}$ is the amplitude of the radar echo of the m-th target after pulse compression, sinc(·) is the sinc function, and *B* is the bandwidth of the signal. In order to facilitate further equation derivation and research, Equation (26) can be abbreviated as:

(27) $$S_{a}\left({\hat{t}}_{a}\right)=\sum_{m=1}^{M}A_{m}\left({\hat{t}}_{a}\right)\cdot \exp\left(-j2\pi f_{a}\tau_{m}\right)$$

In Equation (27), $A_{m}\left({\hat{t}}_{a}\right)=A_{m}\cdot \sin c\left(\pi B\left({\hat{t}}_{a}-\tau_{m}\right)\right)$ is the envelope formed by pulse compression. By combining Equations (23) and (27), we can obtain:

(28) $$S_{a}\left({\hat{t}}_{a}\right)=\sum_{m=1}^{M}A_{m}\left({\hat{t}}_{a}\right)\cdot \exp\left(-j4\pi f_{a}\left(\frac{r_{m}+v_{m}T_{a}}{c}\right)\right)$$

By combining Equations (19), (24) and (28), we can obtain:

(29) $$S_{a}\left({\hat{t}}_{a}\right)=\sum_{m=1}^{M}A_{m}\left({\hat{t}}_{a}\right)\cdot \exp\left(-j4\pi \left(f_{0}+\left(x_{n2}-6.5\right)\cdot \Delta f\right)\cdot \left(\frac{r_{m}+v_{m}\left(T_{0}+\left(x_{n1}-0.5\right)\cdot \Delta T\;\right)}{c}\right)\right)$$

According to Equation (29), the phases of radar echo signals are closely related to chaotic sequences $\left\{x_{n1}\right\}$ and $\left\{x_{n2}\right\}$. As chaotic sequences $\left\{x_{n1}\right\}$ and $\left\{x_{n2}\right\}$ are random sequences, radar echo signals cannot be processed by traditional classical signal processing methods. Through further detailed analysis, Equation (29) can be transformed into:

(30) $$S_{a}\left({\hat{t}}_{a}\right)=\sum_{m=1}^{M}A_{m}\left({\hat{t}}_{a}\right)\cdot \exp\left(-j4\pi \left(f_{0}+\left(x_{n2}-6.5\right)\cdot \Delta f\right)\cdot \frac{r_{m}}{c}+\left(-j4\pi \left(f_{0}+\left(x_{n2}-6.5\right)\cdot \Delta f\right)\right)\cdot \frac{v_{m}\left(T_{0}+\left(x_{n1}-0.5\right)\cdot \Delta T\;\right)}{c}\right)$$

Equation (30) can be further decomposed into:

(31) $$S_{a}\left({\hat{t}}_{a}\right)=\sum_{m=1}^{M}A_{m}\left({\hat{t}}_{a}\right)\cdot \varphi_{rm}\cdot \varphi_{vm}$$

where term $\varphi_{rm}$ is related to the target distance in the echo and called the distance term. The term $\varphi_{vm}$ is related to the target velocity in the echo and called the velocity term.

(32) $$\varphi_{rm}=\exp\left(-j4\pi \left(f_{0}+\left(x_{n2}-6.5\right)\cdot \Delta f\right)\cdot \frac{r_{m}}{c}\right)$$

(33) $$\varphi_{vm}=\exp\left(\left(-j4\pi \left(f_{0}+\left(x_{n2}-6.5\right)\cdot \Delta f\right)\right)\cdot \frac{v_{m}\left(T_{0}+\left(x_{n1}-0.5\right)\cdot \Delta T\;\right)}{c}\right)$$

The anti-sorting signals designed in this paper are PRI and RF multi-parameter composite modulation signals. Therefore, classical signal processing methods are not suitable for designing anti-sorting signals from two aspects of distance and the velocity terms in the phase. The distance term $\varphi_{rm}$ contains the signal carrier frequency term $f_{0}+\left(x_{n2}-6.5\right)\cdot \Delta f$. For the radar with fixed PRI and RF values, $f_{0}+\left(x_{n2}-6.5\right)\cdot \Delta f$ is a fixed value, and the distance term $\varphi_{rm}$ cannot change with the change in any parameters, such as the number of pulses. For a multi-parameter composite modulation anti-sorting signal, the carrier frequency $f_{0}+\left(x_{n2}-6.5\right)\cdot \Delta f$ constantly changes with the change in pulse, so $\varphi_{rm}$ randomly changes with the chaotic sequence $\left\{x_{n2}\right\}$. The velocity term $\varphi_{vm}$ contains the signal carrier frequency term $f_{0}+\left(x_{n2}-6.5\right)\cdot \Delta f$ and the pulse repetition period term $T_{0}+\left(x_{n1}-0.5\right)\cdot \Delta T$. For radar with fixed PRI and RF values, $f_{0}+\left(x_{n2}-6.5\right)\cdot \Delta f$ is a fixed value, $\varphi_{vm}$ values are sampled at equal intervals in the slow time dimension, and the radial velocity of the targets can be measured by fast Fourier transform (FFT). For multi-parameter composite modulation anti-sorting signals, the PRI and RF values jump randomly with the chaotic sequences $\left\{x_{n1}\right\}$ and $\left\{x_{n2}\right\}$, and $\varphi_{vm}$ values are no longer sampled at equal intervals in the slow time dimension, so the velocity cannot be measured by FFT.

Therefore, new radar signal processing technology is needed to process the multi-parameter composite modulation anti-sorting signal.

### 4.2. Target Parameter Estimation Based on CS

According to Section 4.1, the signal echoes in the same distance unit in the slow time dimension cannot be sampled at equal intervals. The target velocity is difficult to measure by traditional classical signal processing methods. Due to the limitation of radar beam width, detection distance and other factors, the number of interest targets in a CPI echo signal is limited. For the LFM signal designed in this paper, matched filter technology is used to match the pulse modulation waveform of the echo signal to achieve meter-level resolution after pulse compression. Furthermore, the echo signal in a distance unit is sparse [43]. Therefore, during radar working, the echo signal meets the requirement of the CS technology for signal sparsity. The actual echo signal always contains noise, so it can be expressed as:

(34) $$S_{a}\left(T_{a}\right)=\sum_{m=1}^{M}A_{m}\left(T_{a}\right)\cdot \exp\left(-j4\pi \left(f_{0}+\left(x_{n2}-6.5\right)\cdot \Delta f\right)\cdot \frac{r_{m}}{c}+\left(-j4\pi \left(f_{0}+\left(x_{n2}-6.5\right)\cdot \Delta f\right)\right)\cdot \frac{v_{m}\left(T_{0}+\left(x_{n1}-0.5\right)\cdot \Delta T\;\right)}{c}\right)+N\left(T_{a}\right)$$

It can be seen from Equation (34) that after pulse compression, the echo signal only contains the distance and the velocity terms and can be expressed as a distance–velocity plane. In order to accurately measure velocity and distance, the unambiguous distance is divided into *S* segments, where *s* is the serial number of the unambiguous distance segment, and s∈{1,2,⋅⋅⋅,S}. The unambiguous velocity is divided into G segments, where *g* is the serial number of the unambiguous velocity segment, and g∈{1,2,⋅⋅⋅,G}. Therefore, the distance–velocity plane is evenly divided into S×G grids, where any grid in the plane represents the corresponding velocity and distance, and $\left(r_{g},v_{s}\right)$ represents the *s*-th range grid within the range represented by the *g*-th range grid. The variables are defined as follows:

(35) $$\begin{array}{l} \varsigma_{s,g}=\alpha_{s,g}\cdot \exp\left(-j4\pi f_{0}\frac{r_{s}}{c}\right)\\ \xi_{s}\left(w\right)=\exp\left(-j4\pi \left(\left(x_{n2}-6.5\right)\cdot \Delta f\right)\frac{r_{s}}{c}\right)\\ \Lambda_{g}\left(w\right)=\exp\left(-j4\pi f_{0}T_{0}\frac{v_{g}}{c}\gamma \left(w\right)\right)\\ \gamma \left(w\right)=\left(1+\left(x_{n2}-6.5\right)\cdot \frac{\Delta f}{f_{0}}\right)\cdot \left(1+\left(x_{n1}-0.5\right)\cdot \frac{\Delta T}{T_{0}}\;\right) \end{array}$$

In Equation (35), *s* and *g* are the segment serial numbers of unambiguous distance and velocity, respectively, and *w* is the pulse number in a CPI. Each pulse signal corresponds to a unique chaotic sequence value to modulate its PRI and RF values. Thus, *w* also corresponds one-to-one to the serial numbers of the chaotic sequences $\left\{x_{n1}\right\}$ and $\left\{x_{n2}\right\}$. Therefore, the echo signal can be expressed as:

(36) $$S_{s,g}\left(T_{a}\right)=\varsigma_{s,g}\xi_{s}\left(T_{a}\right)\Lambda_{g}\left(T_{a}\right)+N\left(T_{a}\right)$$

In Equation (36), $N\left(T_{a}\right)$ is the noise vector. In the following, a dictionary matrix Ψ containing target distance–velocity information is constructed.

(37) $$\Psi ={\left[\Psi_{1},\Psi_{2},\cdot \cdot \cdot ,\Psi_{w}\right]}_{W\times 1}^{T}$$

In Equation (37), $\Psi_{W}$ refers to the submatrix corresponding to the *w*-th pulse in a CPI. $\Psi_{w}$ can be specifically expressed as:

(38) $$\Psi_{W}={\left[\underset{G}{\underset{︸}{\Psi_{11},\Psi_{12},\cdot \cdot \cdot ,\Psi_{1g}}}\cdot \cdot \cdot \cdot \cdot \cdot \underset{G}{\underset{︸}{\Psi_{s1},\Psi_{s2},\cdot \cdot \cdot ,\Psi_{sg}}}\right]}_{1\times \left(S\times G\right)}$$

In Equation (38), $\Psi_{sg}$ can be expressed as:

(39) $$\Psi_{sg}=\exp\left(-j4\pi \frac{s\cdot \Delta r}{c}\left(\left(x_{n2}-6.5\right)\cdot \Delta f\right)\right)\cdot \exp\left(-j4\pi f_{0}T_{0}\frac{g\cdot \Delta v}{c}\gamma \left(w\right)\right)$$

In Equation (39), Δr is the unambiguous distance represented by each segment after the unambiguous distance is equally divided into *S* segments. Δv is the unambiguous velocity represented by each segment after the unambiguous velocity is evenly divided into *G* segments. $x_{n2}$ is the chaotic sequence.

The above is the CS dictionary matrix of the echo signal based on chaotic sequences. Therefore, the CS model of echo signal is expressed as:

(40) y=Ψθ+δ

where δ is the zero-mean measurement noise. According to CS, the problem of target parameter estimation is transformed into the problem of θ reconstruction by target echo measurement value y and matrix Ψ. In radar detection applications, the target usually occupies only a small part of the total distance–velocity coordinate. Therefore, the echo signal can be considered sparse in the distance–velocity domain [44,45]. Then, the original signal is reconstructed by solving an $l_{1}$-norm optimal problem:

(41) $$\langle \hat{\theta }\rangle =\arg\;\min\left({\left\|\theta \right\|}_{1}\right)\;,\;{\left\|y-\Psi \theta \right\|}_{2}\leq \sigma$$

The whole optimization solution process can be divided into two parts. The $l_{2}$-norm is used to ensure the calculation accuracy of the reconstruction results, and the $l_{1}$-norm is used to constrain the sparsity of the reconstruction results. In this paper, the original target signal is sparsely reconstructed by the orthogonal matching pursuit (OMP) algorithm. The OMP algorithm is a greedy algorithm and is an improved matching pursuit (MP) algorithm. The orthogonal operation of the atomic support set is introduced while searching the atomic support set and reserving the MP algorithm. Specifically, the atomic support set of the observed signal is orthogonalized in each cycle in the OMP calculation. Therefore, the OMP algorithm has higher signal recovery accuracy than the MP algorithm. The OMP algorithm has not been improved in this paper, and it is the same as the classic OMP algorithm, so it will not be described here.

## 5. Simulation and Analysis

### 5.1. Simulation of the Signal Design Principle

#### 5.1.1. Correctness of the Signal Design Principle

**Figure 8:**
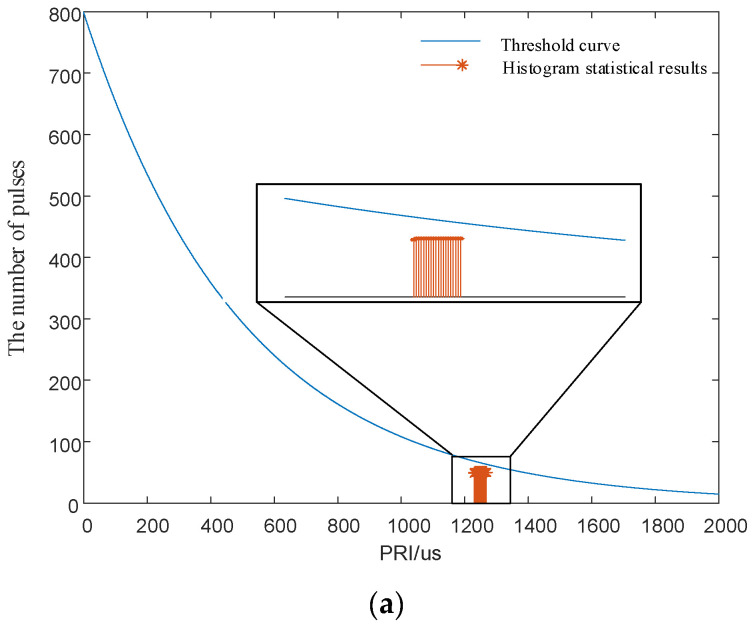

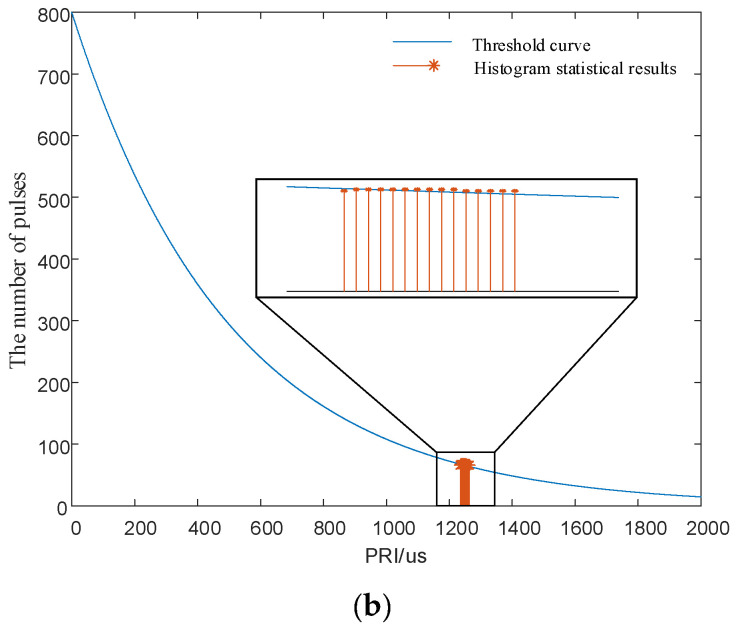
Simulation verification diagrams of the signal design principle. (**a**) First-order TOA difference histogram with a PRI interval length of 20 times minimum interval. (**b**) First-order TOA difference histogram with a PRI interval length of 15 times minimum interval.

The signal design principle shows that when the designed radar signal PRI values follow the interval distribution of which the length is more than 20 times the minimum interval of PRI values, the signal sorting fails. Therefore, the PRI interval length should be at least 20 times the minimum interval when designing signals. In order to verify the correctness and significance of the signal design principle for designing signals, a comparative simulation was performed. The simulation parameters were set as follows:

Each signal had 1000 pulses, and the measurement error of TOAs was 50 ns. The PRI center value of the signal was 1250 μs, and the minimum interval of the PRI values was 2 μs. The interval lengths of PRI agility were 20 and 15 times the minimum interval, respectively. That is, the PRI intervals were [1230 μs,1270 μs] and [1235 μs,1265 μs]. The analysis range of the first-order histogram was 0 to 2500 μs, and the histogram statistics interval is 0.5 μs. The SDIF statistical threshold is shown in Equation (1). In the Equation, *N* = 5000, *k* = 0.1, *a* = 0.8. The SDIF sorting results are shown in Figure 8.

As shown in Figure 8a, when the interval length of the signal PRI is greater than or equal to 20 times the minimum interval, the pulse statistics corresponding to any PRI values in the interval are smaller than the SDIF threshold, and the SDIF algorithm sorting fails. In addition, Figure 8a also shows that the signals designed in this paper still have anti-sorting ability when the PRI jitter amplitude is only 1.6%. As shown in Figure 8b, when the interval length of the signal PRI is less than 20 times the minimum interval, the individual signal pulse statistics in the interval exceed the threshold, and the signal can be successfully sorted by the signal sorting algorithm. This simulation verifies the correctness of the signal design principle and proves that the design principle provides strong theoretical support for designing anti-sorting signals.

#### 5.1.2. Simulation of the Signal Anti-Sorting

**Figure 9:**
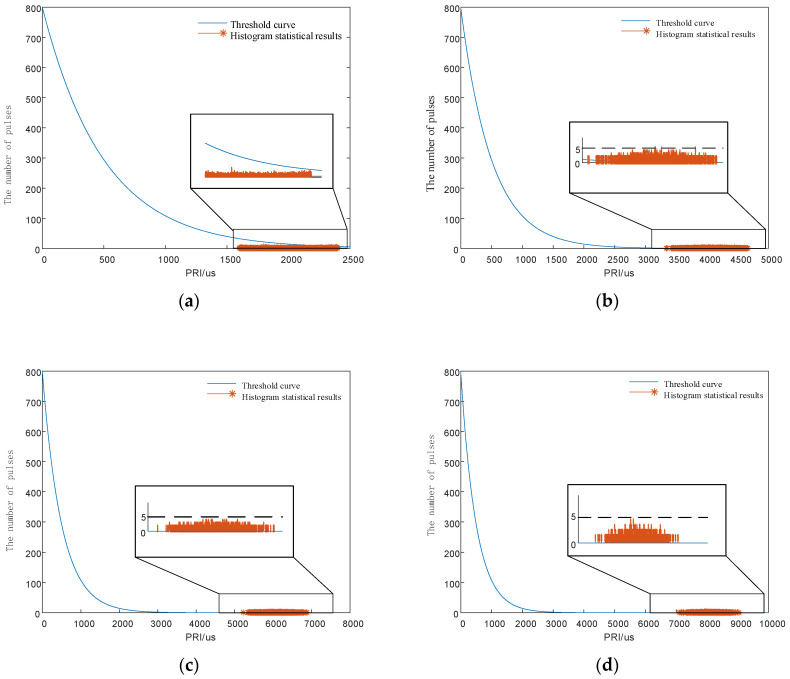
First-order to fourth-order TOA difference histograms. (**a**) First-order TOA difference histogram. (**b**) Second-order TOA difference histogram. (**c**) Third-order TOA difference histogram. (**d**) Fourth-order TOA difference histogram. As shown in (**a**), in the first-order SDIF sorting algorithm, no signal pulses exceed the threshold, so anti-sorting can be achieved. (**b**–**d**) show that although there are signal pulses exceeding the threshold in the second-order, third-order and fourth-order SDIF sorting algorithms, the cumulative number is not more than five, so the signal pulses will not enter the sorting process. Comprehensive analysis of (**a**–**d**) show that the signals modulated by the proposed chaotic map have an anti-sorting effect.

The simulation parameters were set as follows: 

Each signal had a total of 1000 pulses, and the measurement error of TOAs was 50 ns. The PRI center value of the signal was 2 ms, and the variation range of the PRI values was 1000 μs. The analysis range of the first-order histogram was 0 to 2500 μs, and the histogram statistics interval was 0.5 μs. The SDIF statistical threshold is shown in Equation (1). In the Equation, *N* = 5000, *k* = 0.1, *a* = 0.8. The SDIF sorting results are shown in Figure 9.

#### 5.1.3. Comparison and Simulation of the Anti-Sorting Performance of PRI Random Jitter Signals

**Figure 10:**
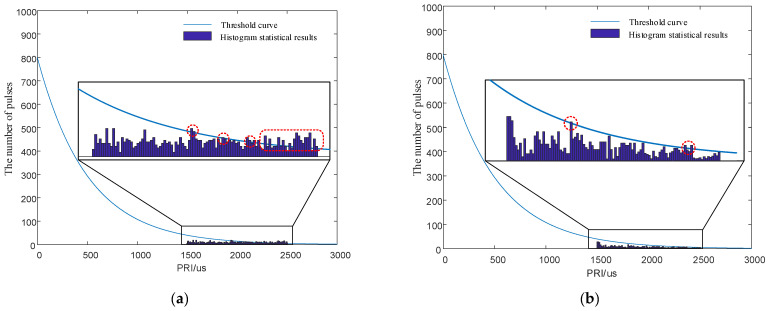
Comparison simulation diagrams of anti-sorting performance. (**a**) First-order TOA difference histogram of the random jitter signals. (**b**) First-order TOA difference histogram of the designed signals.

In the field of signal sorting resistance, PRI random jitter signals are also considered to be highly resistant to sorting. In this section, the anti-sorting ability of PRI random jitter signals and that of the signals designed in this paper were compared. The PRI center value of the jitter signals was 2 ms, and the jitter amount was 1 ms. That is, the PRI interval of the jitter signals was [1500 μs,2500 μs]. For the PRI random jitter signals, PRI values followed a random distribution in the interval [1500 μs,2500 μs]. The signals designed in this paper had the PRI generated by chaotic sequence modulation in the interval [1500 μs,2500 μs]. The signals to be sorted had a total of 1000 pulses. The simulation results are shown in Figure 10.

Figure 10a is the SDIF of the PRI random jitter signals. The red circles mark where the histogram statistics exceed the detection threshold of the sorting algorithm. Many places marked by red circles indicate that the signals are easily sorted by the SDIF algorithm. A small number of TOA difference histograms exceed the threshold of the sorting algorithm in Figure 10b. However, the comparison of Figure 10a,b shows that under the guidance of the signal design principle in this paper, only a few signal histograms of the signals modulated by chaotic sequences exceed the threshold. However, a large number of histogram statistics exceed the threshold in the histogram of PRI random jitter signals, indicating that the signals designed in this paper are superior to the PRI random jitter signals in anti-sorting performance.

#### 5.1.4. Quantization Simulation of Signal Anti-Sorting Performance

**Figure 11:**
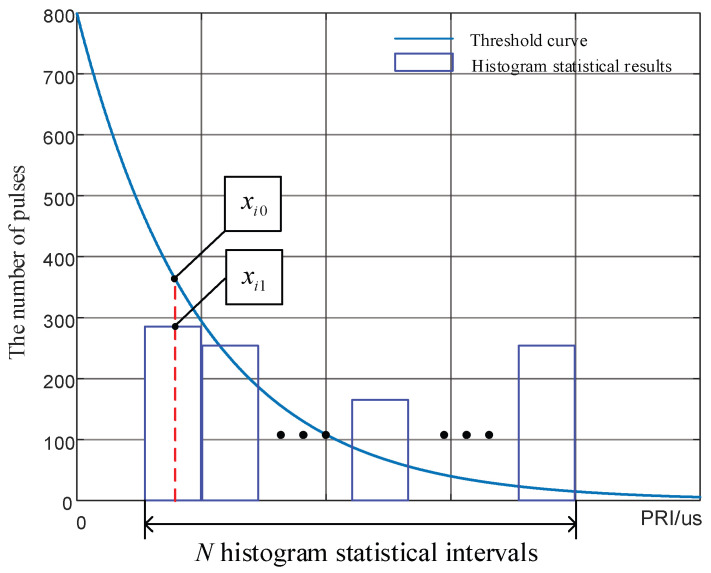
Schematic diagram of margin calculation.

In the past, the anti-sorting ability of signals was measured mainly by the TOA difference histogram method for qualitative analysis, by which the anti-sorting ability of designed signals for the SDIF algorithm cannot be accurately described. To quantify the anti-sorting performance of the RF stealth signals designed in this paper, the concept of “margin” was introduced. In each statistical interval of the histogram, the threshold corresponding to the center PRI of the statistical interval was subtracted from the actual statistical value of the histogram to obtain the margin value of each statistical interval. Then, the margin values of all statistical intervals were summed and averaged to quantify the anti-sorting ability of the signals. The margin values can be calculated using Equation (42), and the schematic diagram of the calculation is shown in Figure 11.

(42) $$Y=\frac{1}{n}\sum_{i=1}^{n}\left(x_{i0}-x_{i1}\right)$$

where $x_{i0}$ is the threshold corresponding to the center PRI of the *i*-th statistical interval, $x_{i1}$ is the actual statistical value of the histogram corresponding to the center PRI of the statistical interval, *n* is the number of statistical intervals, and *Y* is the margin value.

According to the calculation process, a positive margin value illustrates that the statistical value of a signal in a certain interval in the SDIF is below the threshold. A larger margin value indicates a larger distance between the statistical value and the threshold in this interval and better anti-sorting performance of the signal. If the margin value is negative, the statistical value in a certain interval in the SDIF is above the threshold. This phenomenon indicates that the signals in this PRI interval have been sorted by the SDIF algorithm. Using the margin, the anti-sorting performance of the signals can be accurately reflected.

**Figure 12:**
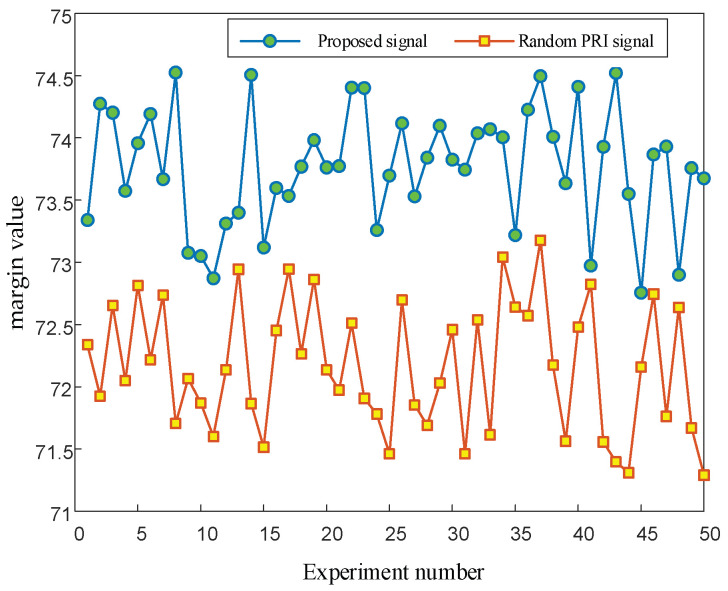
Line chart of signal margin values.

The simulation parameters were set as follows: the signal had a total of 10,000 pulses, and the measurement error of TOAs is 50 ns. The PRI center value of the signal was 2 ms, the PRI variation range was 1000 μs, the analysis range of the first-order histogram was 0 to 2500, and the histogram statistical interval was 0.5 μs. The SDIF statistical threshold is shown in Equation (1). *N* = 5000, *k* = 0.1 and *a* = 0.8. To ensure that the margin values can reflect the anti-sorting performance of the signals more accurately, 1000 Monte-Carlo simulations were performed. Then, 50 of them were taken for drawing (Figure 12).

Figure 12 shows that the margin values of the anti-sorting signals designed in this paper are significantly higher than those of the random PRI signals, indicating that the anti-sorting performance of the signals designed in this paper is significantly higher than that of the random PRI signals.

### 5.2. Performance Simulation of the Chaotic System

#### 5.2.1. Performance Simulation of the Chaotic Mapping

In order to further illustrate the advantages of the chaotic map proposed in this paper, the typical one-dimensional chaotic maps, including the Tent, logistic, Chebyshev, and cubic maps, were selected and compared with the proposed map. We analyzed and compared the bifurcation diagrams, maximum Lyapunov exponents, approximate entropies and balances of the chaotic maps.

**Figure 13:**
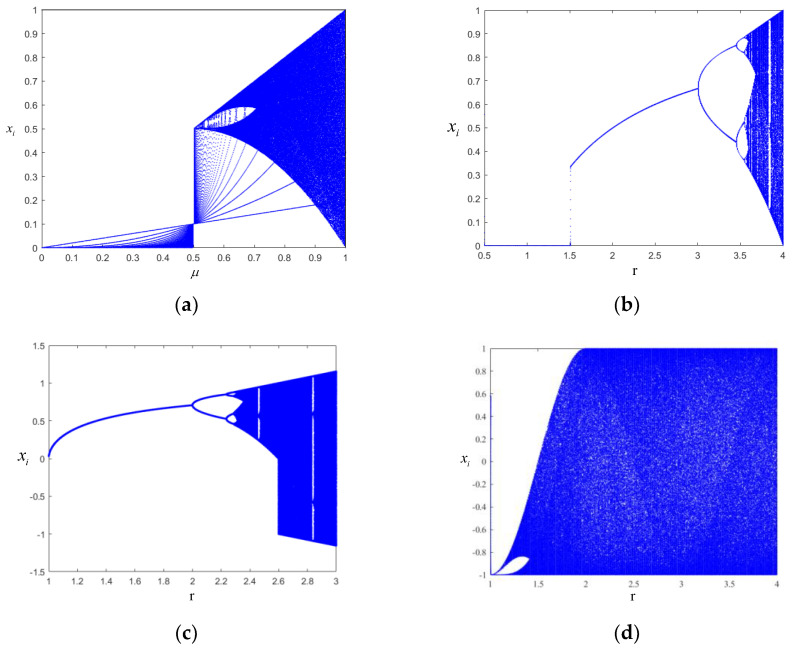

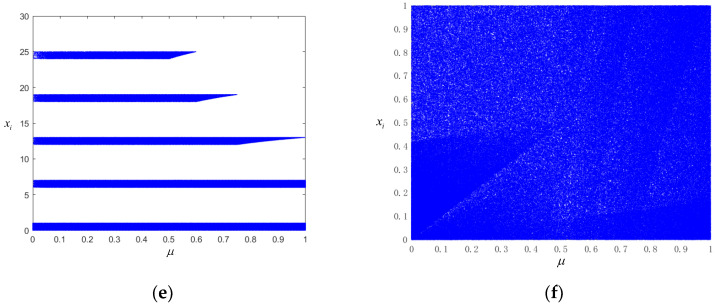
Bifurcation diagrams of the five chaotic mappings. (**a**) Bifurcation diagram of the Tent chaotic mapping. (**b**) Bifurcation diagram of the logistic chaotic mapping. (**c**) Bifurcation diagram of the cubic chaotic mapping. (**d**) Bifurcation diagram of the Chebyshev chaotic mapping. (**e**) Bifurcation diagram of the SNP-PLCM. (**f**) Bifurcation diagram of the SNP-PLCM (An enlarged view of the first sequence point set in (**e**).

The bifurcation of a chaotic map is one of the signs that the map has entered a chaotic state. By describing the bifurcation diagrams of the maps, we can directly observe the chaotic regions of the maps and the influences of the control parameters in the map on chaos. The bifurcation diagrams are drawn according to the expressions of the five mappings, namely the logistic, Chebyshev, cubic, and Tent mappings and the mapping proposed in this paper (Figure 13).

As shown in Figure 13, only when the fractal coefficient of the Tent mapping μ>0.7, the system can enter a chaotic state; when 0<μ<0.7, the system is in a periodic state. When the fractal coefficient of the logistic mapping r≥3.57, the system is in a chaotic state; when 0≤r<3.57, the system is in a periodic state. As for the cubic mapping, when the fractal coefficient r≥2.3, the system is in a chaotic state; when 0≤r<2.3, the system is in a periodic state. When the fractal coefficient of the Chebyshev mapping r≥1.5, the system can be in a chaotic state; when 0≤r<1.5, the system is in a periodic state. The chaotic mapping proposed in this paper is not affected by the fractal coefficient, and the points in the bifurcation diagram are more uniformly distributed.

Lyapunov exponents (*LE*s) can characterize the motion characteristics of a chaotic system. The positive, negative and magnitude of an *LE* value in a certain direction represent the average divergence $\left(\lambda_{i}>0\right)$ or convergence $\left(\lambda_{i}<0\right)$ rate of adjacent orbits in the attractor of a long time system in this direction. Therefore, the minimum *LE* $\lambda_{\min}$ determines how fast the orbit converges, and the maximum *LE* $\lambda_{\max}$ determines how fast the orbit covers the whole chaotic attractor. The maximum *LE* is used to evaluate the randomness of chaotic sequences, and its definition is shown in Equation (43):

(43) $$LE=\underset{n\to \infty }{\lim}\left(\frac{1}{n}\sum_{i=0}^{n-1}\ln\left|f^{\prime }\left(x_{i}\right)\right|\right)$$

where $f^{\prime }\left(x_{i}\right)$ represents the first derivative of the chaotic map $f\left(x_{i}\right)=x_{n+1}$. When LE>0, the system is in a chaotic state, and a larger value indicates stronger randomness of the chaotic sequence produced by the system. The *LE* change curves of the above five chaotic maps with different fractal coefficients are drawn as follows.

**Figure 14:**
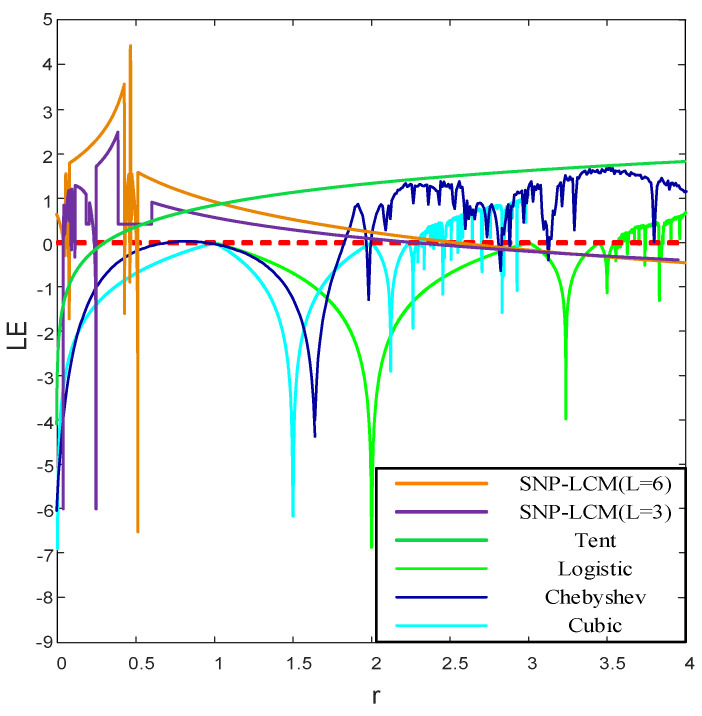
Comparison of the *LE*s of different chaotic mappings.

According to Figure 14, the *LE*s of the Tent, logistic, Chebyshev and cubic mappings are not always greater than 0, indicating that the four systems are not always in a chaotic state. Taking the sky-blue *LE* change curve of the cubic mapping in the figure as an example, we can observe that only when the fractal coefficient r>2.3, the *LE* is greater than 0, indicating that the system is in a chaotic state. Compared with other classical one-dimensional chaotic maps, the chaotic map designed in this paper has higher *LE*s in the variation interval of fractal coefficients, indicating that the chaotic sequences generated by the proposed SNP-PLCM have stronger randomness than the chaotic sequences generated by the other four one-dimensional chaotic maps. The analyses of *l* = 3 and *l* = 6 curves show that a larger interval number selection parameter of the SNP-PLCM designed in this paper leads to stronger randomness of the sequences generated by chaotic mapping.

Approximate entropies can be used to measure the complexity of the sequences generated by chaotic maps. A larger approximate entropy indicates the higher complexity of a sequence. The approximate entropies of the above four mappings were calculated under the same simulation conditions. The fractal coefficients varied from 0.5 to 4 in steps of 0.5, and the sequence length was set to 10,000. The results are shown in Table 1. When the system does not enter a chaotic state, the value of approximate entropy is extremely small or even 0. For example, when r=0.5 and r=1, the approximate entropies of the logistic and cubic maps are at the order of $10^{-5}$ or $10^{-7}$. When r=1.5, the approximate entropy of the cubic map is 0. When r=1, the approximate entropy of the Chebyshev map is 0. However, the SNP-PLCM designed in this paper maintains an approximate entropy greater than 1 or close to 1 in the variation range of the fractal coefficients of (0,4], showing that the sequence generated by it has higher complexity.

Fourth, the recursion graph is a non-stationary signal processing and analysis method. It has been widely applied in the analysis of chaotic signals and chaotic signal modulation based on chaos. The recursion graph is plotted by the recursive matrix, which can be expressed by Equation (44):

(44) $$R_{i,j}\left(\varepsilon \right)=\Theta \left(\varepsilon -\left\|{\vec{\eta }}_{i}-{\vec{\eta }}_{j}\right\|\right)\;i,j=1,2,\cdot \cdot \cdot ,N$$

where $R_{i,j}$ is a square matrix of N×N; N is the number of state vectors ${\vec{\eta }}_{i}$; the threshold ε represents a preset critical distance; ‖·‖ is the norm; Θ(·) is the unit step function, Θ(x<0)=0, Θ(x>0)=1. A recursion graph is obtained by drawing a recursive matrix with different colors to represent its binary. The chaotic signal is analyzed through the recursion graph to obtain the law of the chaotic signal. Periodic or quasi-periodic recursive structures (e.g., checkerboard-like structures) appear in the diagonal direction, and these structures characterize the periodicity of the signal state evolution. If the recursion graphs are all isolated recursive points, and the isolated recursive points follow a uniform distribution with almost no relatively long diagonals, vertical lines, or horizontal lines, it indicates good randomness of the signal. The recursion graphs of the sine sequence sin(2πt), logistic, cubic, Tent, and the sequence generated by the chaotic map are shown below, with 3,100 sequence point values for each sequence.

**Figure 15:**
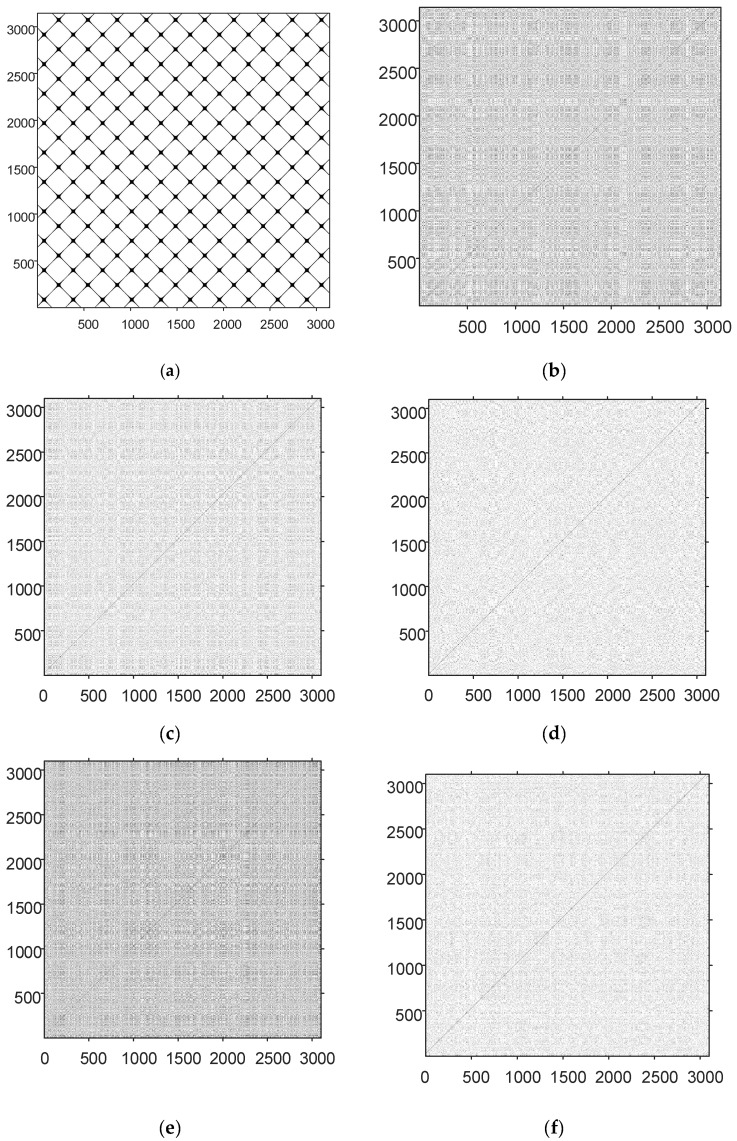
The RP of different sequences. (**a**) Recursion graph of sin sequence; (**b**) Recursion graph for logistic sequences; (**c**) Recursion graph of cubic sequences; (**d**) Recursion graph of Tent sequences; (**e**) Recursion graph of SNP-PLCM sequences (L = 3); (**f**) Recursion graph of SNP-PLCM sequences (L = 6).

As shown in Figure 15a, the recursion graph of the sinusoidal sequence presents an obvious grid-like recursion structure, indicating that the sinusoidal sequence has a significant periodicity. The recursion graphs of (b) logistic, (c) cubic, and (d) Tent sequences have no significant recurrence structure. The distribution of recurrence points is not uniform, and there are still some short lines, indicating the phenomenon of short periods in the sequence. Figure 15e,f are the recursion graphs of the designed mappings when the interval parameters are 3 and 6, respectively. As shown in Figure 15e, when the interval parameter is 3, the uneven distribution of recurrence points also partially emerges in the recurrence graph, with a small grid-like recurrence structure. In Figure 15f, when the interval parameter is 6, there is only one 45° main diagonal line, and the rest of the recursion points are fully scattered and uniformly distributed in the graph. In addition, the distribution of the local recursion points is consistent, indicating that the chaotic sequence in this study has strong randomness. 

Fifth, the recursion graphs are mainly for the qualitative analysis of chaotic signals. Therefore, recursive quantitative analysis is still needed for quantitative analysis and comparison of chaotic sequences. According to the literature [46], the recursive rate (RR), entropy (ENTR), system determinacy (DET), and maximum diagonal length ($L_{\max}$) are mainly used to analyze chaotic sequences. The larger recurrence rate indicates a more concentrated distribution of points in the recursive graph of the system. In contrast, the lower recurrence rate indicates a more uniform distribution of points in the recurrence graph of the system. With higher entropy, the system is more complex, while lower entropy indicates a less complex system. Furthermore, a larger DET value indicates that the chaotic sequence is more deterministic, while a smaller DET value indicates that the chaotic sequence is more random. In the phase space, the faster divergence of the attractor trajectory leads to a shorter maximum diagonal.

It can be seen from Table 2 that the chaotic map with an interval parameter of 6 has the lowest recurrence rate, indicating that all points of the map are uniformly distributed without aggregation, consistent with the recursion graph in Figure 15. Regarding system certainty, the proposed chaotic map has the smallest DET value when the interval parameter is 6, which is close to the DET value of white Gaussian noise, indicating that the proposed chaotic map has strong randomness. In terms of maximum diagonal length, the proposed chaotic map has an extremely short diagonal length. The diagonal length of the SNP-PLCM map with interval parameter 6 is approximately the same as that of Gaussian white noise. As a result, the attractor trajectory of the proposed chaotic map diverges rapidly in the phase space. Finally, the chaotic map designed in this paper has the largest entropy, much larger than logistic, cubic, and Tent maps, suggesting that the proposed chaotic map is relatively complex. 

#### 5.2.2. Signal Parameter Sequence Performance Analysis

**Figure 16:**
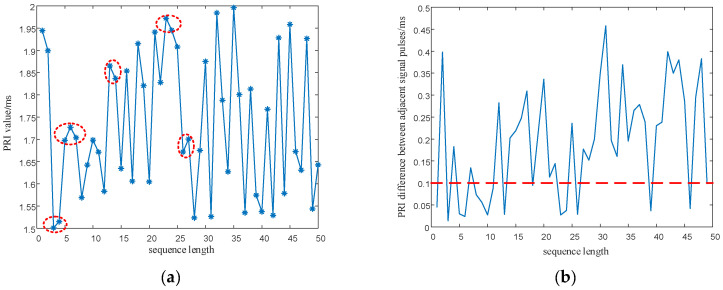
PRI sequence and PRI difference between adjacent signals before optimization. (**a**) PRI sequence before optimization; (**b**) PRI difference between adjacent signals before optimization.

**Figure 17:**
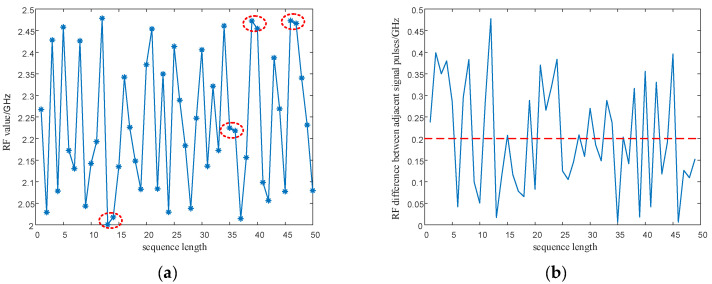
PRI sequence and RF difference between adjacent signals before optimization. (**a**) RF sequence before optimization; (**b**) RF difference between adjacent signals before optimization.

It can be seen from Figure 16 and Figure 17 as the red circle and the red dash lines that if the proposed SNP-PLCM chaotic map is directly used to modulate the signal parameters, it can lead to the multi-value narrow interval effect of the parameters. The multi-value narrow spacing effect can easily cause the modulated signal PRI to fall within the same sorting tolerance, which is not conducive to the anti-sorting signal performance. Therefore, a design method for wide-spaced signals is proposed in Section 3.2. The wide-interval signal design method is further examined by simulation, and the specific parameters are shown in Table 3. 

**Figure 18:**
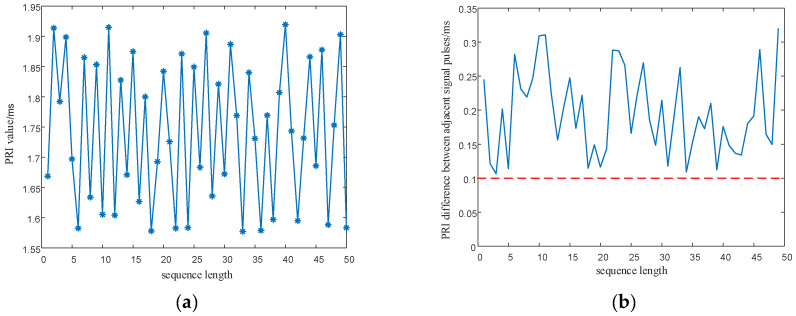
PRI sequence and PRI difference between adjacent signals after optimization. (**a**) Optimized PRI sequence; (**b**) PRI difference between adjacent signals after optimization.

**Figure 19:**
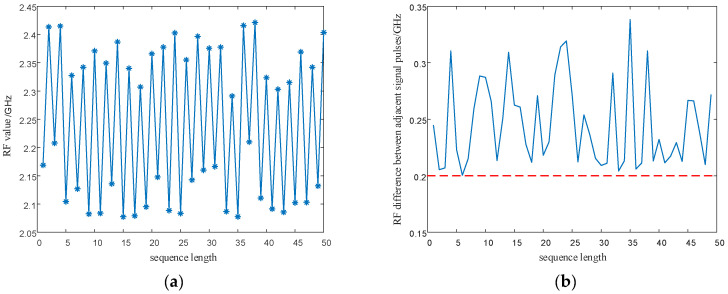
RF sequence and RF difference between adjacent signals after optimization. (**a**) Optimized RF sequence; (**b**) RF difference between adjacent signals after optimization.

In order to eliminate the initial value effect of the chaotic system on the modulation of signal parameters, the chaotic sequence is firstly iterated 500 times, with modulation starting from the 501st time. In addition, the length of the chaotic sequence is set to 50 to observe the modulated parameter values of the chaotic sequence. The simulation results are shown in Figure 18 and Figure 19. 

As shown in Figure 18 and Figure 19, the proposed method optimizes the generation method of PRI and RF sequences, eliminates the effect of multi-value narrow intervals, and satisfies the requirement of PRI and RF difference intervals. In addition, the proposed method enhances the anti-sorting performance of the signal. After analyzing the simulation results in Figure 16, Figure 17, Figure 18 and Figure 19, the flexibility of the designed signals can be explained. Using the chaotic sequence to modulate the PRI and RF of the signal can fully utilize the initial value sensitivity and fractal coefficient sensitivity of the chaotic system, providing a wider variation range for the signal PRI and RF values. Under different working conditions, the chaotic sequence offers greater flexibility in the design of signal PRI and RF. According to the total length of the designed signal, PRI and RF sequences can be generated at one time or divided into several segments and generated multiple times. The PRI and RF sequences obtained in this way are also different.

### 5.3. Signal Parameter Estimation Based on CS

#### 5.3.1. Reconstruction and Recovery of LFM Signals

**Figure 20:**
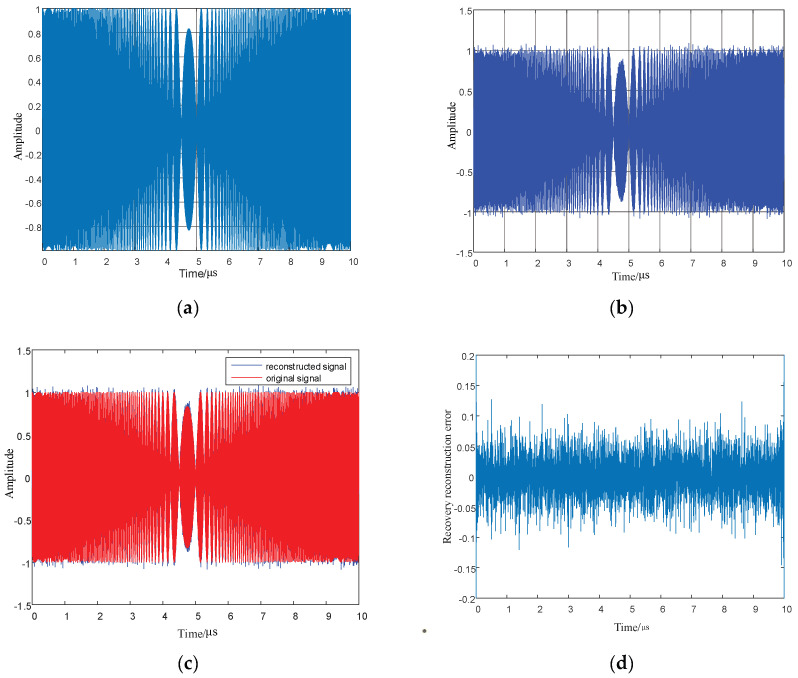
Original signal in linear frequency modulation time domain, reconstructed signal, and error diagram. (**a**) Original signal in linear frequency modulated time domain; (**b**) Reconstructed signal in linear frequency modulation time domain; (**c**) Original signal and reconstruction signal in linear frequency modulation time domain; (**d**) Error between the original signal and reconstructed signal in linear frequency modulation time domain.

**Figure 21:**
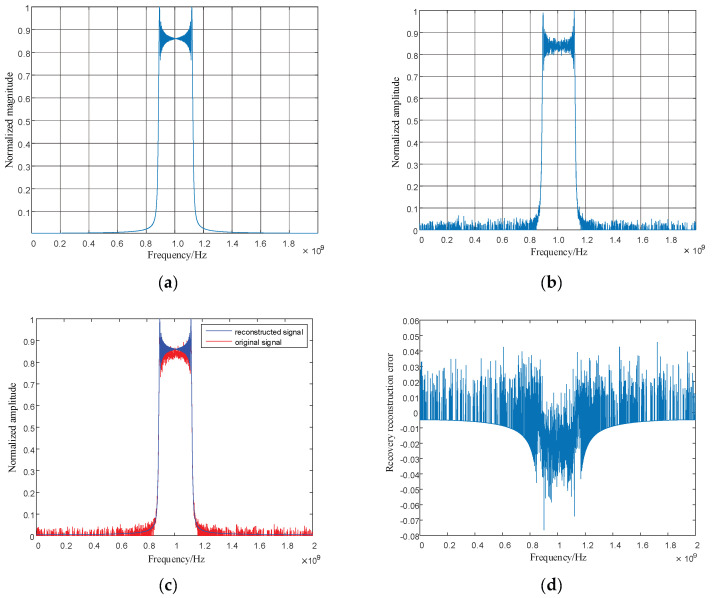
Original signal in the linear modulation frequency domain, reconstructed signal, and error diagram. (**a**) Original signal in the linear modulation frequency domain; (**b**) Reconstructed signal in the linear modulation frequency domain; (**c**) Original signal and reconstructed signal in the linear modulation frequency domain, (**d**) Error between the original signal and reconstructed signal in the linear modulation frequency domain.

Parameter settings: The initial carrier frequency of the LFM is 1 GHz; the signal bandwidth is 220 MHz; the pulse width is 10 microseconds; the signal sampling time is 10 microseconds; the sampling points are 4096 points; the CS measurement points are 1024 points. The Orthogonal Matching Pursuit (OMP) algorithm is used to recover and reconstruct the LFM signal. Detailed simulation results are shown in Figure 20 and Figure 21.

As shown in Figure 20 and Figure 21, LFM signals in the time domain and frequency domain are recovered to verify the recovery performance of CS technology on LFM signals. Furthermore, the original signal and the reconstructed signal are plotted in a simulation diagram, and the error between the original and reconstructed signals in the time domain and frequency domain are calculated. It can be seen from Figure 20d that when the LFM time domain signal is recovered using the CS technology, the overall recovery reconstruction error is less than 10%, and the recovery reconstruction error reaches 20% at the beginning and the end of the signal. Figure 21d shows that the linearly tuned domain signal using compression perception technology is frequently recovered, and the recovery error is within ±10%. This result demonstrates that the compression perception technology of the linear frequency modulation time domain and frequency domain signals has good recovery reconstruction performance, reducing the signal measurement points and achieving accurate signal recovery.

#### 5.3.2. The Processing of LFM Signals

In this subsection, the signal processing algorithm in Section 4 is verified by simulation. The specific simulation parameters are shown in Table 4. 

According to the parameter settings, the maximum unambiguous distance of the signal is 150 km, and the maximum unambiguous speed is 50m/s. Since there are 64 segments in both the unambiguous distance and speed, the unambiguous distance represented by each segment is 2.3438 km, and the unambiguous speed represented by each segment is 0.7813 m/s. In the simulation, the distance between the target and the radar is assumed to be 65 km, and the radial velocity is 12 m/s. The CS technology is used in the simulation design to process the echo signals, and the simulation results are shown in Figure 18.

**Figure 22:**
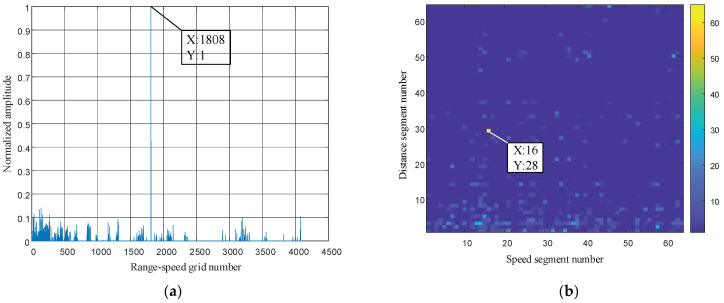
The target-range velocity recovered and reconstructed by the CS algorithm. (**a**) One-dimensional graph of the recovery and reconstruction of target-range velocity; (**b**) Two-dimensional graph of the recovery and reconstruction of target-range velocity.

It can be seen from the figure that the range and velocity of the target are obtained through CS technology to reconstruct and restore the echo signal. In Figure 22b, the speed and distance of the target are intuitively displayed. The velocity of the repaired and reconstructed target is 16×0.7813=12.5008 m/s, and the distance is 28×2.3438=65.6264 km, which is very close to the parameter settings of the target. 

#### 5.3.3. Analysis of Influencing Factors

In this section, the factors affecting signal reconstruction are analyzed through simulation, mainly including the signal compression factor and SNR. In order to intuitively illustrate the influence of the compression factor and SNR on signal reconstruction error, the root mean squared error (RMSE) is used as the measurement standard, as shown in Equation (45):

(45) $$R=\sqrt{\frac{1}{n}\sum_{i=1}^{n}{\left({x^{\prime }}_{i}-x_{i}\right)}^{2}}$$

where ${x^{\prime }}_{i}$ is the point value after restoration and reconstruction; $x_{i}$ is the point value corresponding to the original signal; *n* is the total number of signal points.

(1) The compression ratio.

**Figure 23:**
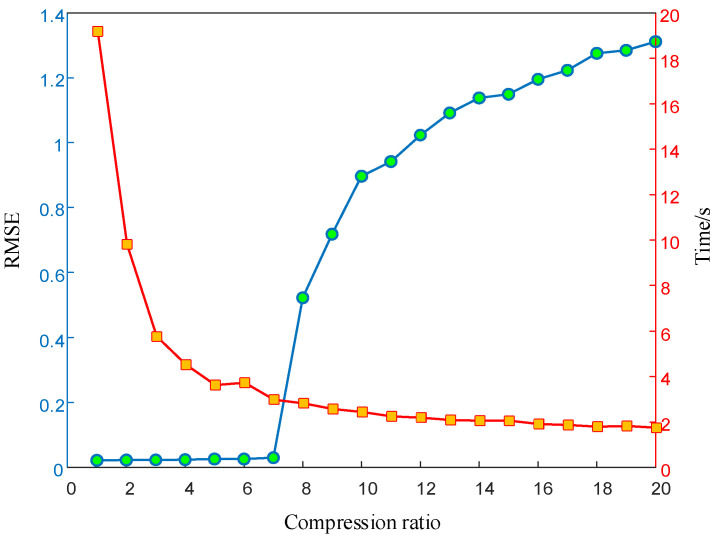
The RMSE of the reconstructed signal and the relationship between the reconstructed time and CS compression ratio.

In order to study the influence of different compressive sensing multiples on signal reconstruction and recovery, the compression multiples sequence $\left\{x|x\in \left[1,20\right],x\in N^{+}\right\}$ of the CS algorithm is simulated. Parameter setting during simulation: The initial carrier frequency of the LFM signal is 1 GHz; the signal bandwidth is 50 MHz; the amplitude is 1; the pulse width is 10 microseconds; the signal sampling time is 10 microseconds; the number of pulse signals is 4,096 points; and the ideal state is no noise interference. The OMP algorithm is used to restore and reconstruct the LFM signal, and the simulation results are shown in Figure 23.

The results in Figure 23 are analyzed from two dimensions. In terms of recovery and reconstruction accuracy (the blue curve in the figure), in general, the error in time-domain recovery and reconstruction of LFM signal increases with the increase of the compression factor, indicating that the reconstruction error increases with the decrease of CS algorithm measurements. During the change of the compression factor from one to seven times, the change of the RMSE has little fluctuation and stays within 0.1. When the compression factor starts from eight times, the RMSE increases significantly because the number of measurements is less at the critical value of the minimum number of measurements. In terms of the recovery and reconstruction time of the CS algorithm (the red curve in the figure), the time decreases with the increase of the compression factor. When the compression factor changes from one to five, the time significantly decreases, demonstrating the advantage of CS technology in shortening the signal processing time. Based on these results, the optimal reconstruction accuracy and time can be obtained when the compression factor is seven times.

(2) SNR.

**Figure 24:**
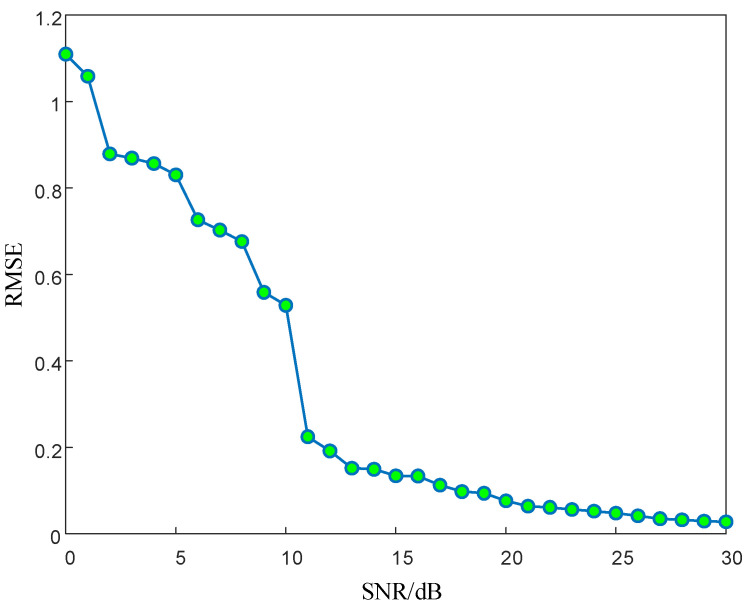
Relationship between RMSE and SNR of reconstructed signal in linear frequency modulation time domain.

In order to study the influence of SNR on signal reconstruction and recovery, the SNR sequence $\left\{x|x\in \left[0,30\right],x\in N^{+}\right\}$ is simulated in this subsection. The simulation parameters are set as follows: The initial carrier frequency of the LFM signal is 1 GHz; the signal bandwidth is 50 MHz; the amplitude is 1; the pulse width is 10 microseconds; the signal sampling time is 10 microseconds; the number of pulse signal points is 4,096; the number of measurement points is 1,024; and noise interference exists. The OMP algorithm is used to restore and reconstruct the LFM signal, and the simulation results are shown in Figure 24. 

As shown in Figure 24, the recovery reconstruction error gradually decreases with the increase of SNR. When the SNR increases to 10 dB, the RMSE significantly decreases from 0.58 to 0.2. With the continuous increase of SNR, the decrease in error gradually slows down. When the SNR is 30 dB, the RMSE decreases to 0.027.

## 6. Conclusions

In order to enhance the anti-sorting ability of radar RF stealth and realize the detection of targets by radar, this study investigates the principle of SDIF sorting failure and proposes the corresponding signal design method. Afterward, the PRI signal is modulated with the SNP-PLCM chaotic system to obtain a PRI wide-interval anti-sorting signal. To further enhance the anti-sorting performance of the signal in practical engineering applications, the RF and PRI of the signal are usually designed to jump simultaneously. Moreover, an anti-sorting signal processing method based on CS is proposed to solve the velocity and distance problems of the target. Simulation results show that the signal has strong flexibility to meet the requirements of anti-sorting. It improves the anti-sorting performance and enables the study of signal design and signal processing. The findings of this study provide an important basis for the design and engineering implementation of anti-sorting signals.

## Figures and Tables

**Table 1:** The approximate entropy of five different chaotic mappings varies with the fractal coefficient.

r	Logistic	Cubic	Chebyshev	Proposed
0.5	7.2584 × 10^−7^	8.2199 × 10^−7^	2.7641 × 10^−5^	1.9331
1	2.0764 × 10^−5^	2.9270 × 10^−5^	0	1.5572
1.5	7.1890 × 10^−7^	0	0.6103	1.6901
2	1.8422 × 10^−7^	0.0107	0.7113	1.6842
2.5	8.2893 × 10^−7^	0.60	0.8543	1.2943
3	0.0111	1.1301	1.0146	1.0305
3.5	0.0016	0	1.0976	1.0002
4	0.70	0	1.2311	0.9862

**Table 2:** Recursive quantitative analysis of different sequences.

Sequence Types	RR	DET	$L_{\max}$	ENTR
sin(2πt)	0.0820	0.7787	3079	0.2284
White Gaussian noise	0.0561	0.1088	5	2.3611
Logistic	0.0770	0.7003	23	0.3611
Cubic	0.0822	0.6703	12	0.4356
Tent	0.0799	0.6059	16	0.4123
SNP-PLCM (L = 3)	0.0648	0.3758	11	1.2515
SNP-PLCM (L = 6)	0.0564	0.2737	7	1.5422

**Table 3:** Signal simulation parameter setting.

Serial Number	Parameter Type	Center Value	Parameter Variation	Tolerance	Minimum Parameter Interval between Adjacent Pulses
1	PRI	1.5 ms	1000 μs	50 μs	100 μs
2	RF	2000 MHz	1000 MHz	100 MHz	200 MHz

**Table 4:** Simulation parameter settings.

Simulation Parameter	Value	Simulation Parameter	Value
Center carrier frequency/GHz	3	Pulse repetition period/ms	1
Maximum variation of carrier frequency/GHz	0.3	The maximum change in pulse repetition period/ms	0.1
Bandwidth/MHz	50	Pulse width/μs	200
Number of pulses in a CPI	64	Signal-to-noise ratio (SNR)/dB	30
Mean segment number of unambiguous distance	64	Mean segment number of unambiguous speed	64
Simulation time/ms	64		

## Data Availability

Not applicable.

## Abbreviations

RF	Radio frequency
SDIF	Sequential difference histogram
CS	Compressed sensing
PW	Pulse width
DOA	Direction of arrival
TOA	Time of arrival
PRI	Pulse repetition interval
SNP-PLCM	Segment number parameter-piecewise linear chaotic map
CDIF	Cumulative difference histogram
RR	Recursive rate
ENTR	Entropy
DET	Determinacy
*L*_max_	Maximum diagonal length
OMP	Orthogonal Matching Pursuit
LFM	Linear frequency modulation
RMSE	Root mean squared error
SNR	Signal-to-noise ratio
